# Theoretical Investigation of Structural and Optical Peculiarities of Bikaverin Fungal Pigment in Chloroform Solution

**DOI:** 10.3390/molecules30234634

**Published:** 2025-12-02

**Authors:** Anastasia Povolotckaia, Dmitrii Pankin, Sergey Belousov, Andrey Boyko, Sergey Akulov, Evgenii Borisov, Anatoliy Gulyaev, Sergey Gudkov, Andrey Izmailov, Maxim Moskovskiy

**Affiliations:** 1 Federal Scientific Agroengineering Center VIM, 109428 Moscow, Russia; 2 Center for Optical and Laser Materials Research, St. Petersburg State University, 198504 St. Petersburg, Russia; 3 Department of Processes and Machines in Agribusiness, Kuban State Agrarian University Named After I.T. Trubilin, 350044 Krasnodar, Russia; 4 Don State Technical University, 346780 Rostov-on-Don, Russia; 5 Prokhorov General Physics Institute of the Russian Academy of Sciences, 119991 Moscow, Russia; 6 Bauman Moscow State Technical University, 5 2nd Baumanskaya St., 105005 Moscow, Russia

**Keywords:** bikaverin, density functional theory, conformer, tautomer, vibrational properties, UV-vis absorbance, *Fusarium* fungi pigment

## Abstract

Bikaverin is a polyketide pigment metabolite produced by certain *Fusarium* genus fungi. It has a number of promising applications due to its biological properties, including a cytotoxic effect against certain cancer cell lines, antioomycete and nematicidal biological activity, and potential antiviral activity. A more accurate structural characterization using quantum chemical calculation methods may facilitate the study of other useful biological properties. Therefore, in this study, a single-molecule density functional theory study of bikaverin in chloroform solvent was conducted. In addition to the lowest state, two rotational conformers with energies higher by 0.74 and 0.32 kcal/mol were found, as well as the presence of a stable low-energy tautomeric state higher in energy by 1 kcal/mol. IR absorption spectra and UV-visible electronic absorption spectra were modeled for certain states. The attribution of the observed spectral peculiarities was performed. Vibrational modes and peaks sensitive to structural peculiarities were proposed for the IR absorption spectra of various energy states. The results obtained can be used to further control the structure of bikaverin and its derivatives.

## 1. Introduction

Bikaverin (CAS No. 33390-21-5, 7,10-dihydro-6,11-dihydroxy-3,8-dimethoxyl-methyl-12*H*-benzo[b]xanthene-7,10,12-trione [1]) occupies a special place among the naphthoquinone pigments. Initially isolated from *Gibberella fujikuroi* (a teleomorphic form of *Fusarium fujikuroi*) in 1957 [2,3], this pigment was subsequently also isolated from a number of fungi of the genus *Fusarium*, including *Fusarium oxysporum*, *F. verticillioides*, *F. fujikuroi* and *F. proliferatum* [4,5,6].

The practical significance of this pigment research is associated with both fundamental reasons, i.e., studying the genes in fungi that synthesize it and establishing biosynthesis pathways in various fungi [6,7,8,9,10], and a number of applied reasons mainly in pharmaceutics for designing new drugs [10].

From the very beginning of its research to the present day, a particular interest in studying the biological activity of this polyketide pigment arose, with perspective applications in medicine, pharmacology and agriculture. Previous studies investigating its biological properties have noted the following features. Bikaverin exhibits a cytotoxic effect against certain cancer cell lines, including sarcoma, leukemia, lymphoma and Ehrlich carcinoma [8,11,12]. Furthermore, bikaverin has demonstrated antimicrobial and antiprotozoal effects [4,10,13]. Additionally, theoretical studies using molecular docking have demonstrated its potential as an antiviral candidate for the treatment of COVID-19 [14]. The applied significance of bikaverin research is also related to agriculture. This pigment exhibits antioomycete and nematicidal biological activity [15,16]. Moreover, this pigment, being a metabolite of certain fungi, can be treated as an indicator of infection by the type of fungi [17]. Another area of possible pigment application is textile industry where it can be used as a blue pigment [18].

The bikaverin molecular structure was investigated and approved using several experimental techniques: NMR spectroscopy [19,20,21], UV-visible (UV-vis) absorbance spectroscopy [8,20,22], IR absorbance spectroscopy [19,20,21,22], liquid chromatography [17,23] and mass spectrometry [17,19]. To the authors’ best knowledge, only one article devoted to bikaverin in solid state and its single crystal XRD study [1] is presented. Moreover, very limited number of theoretical papers on bikaverin could be found; for example, investigation of bikaverin as a biosensor using a density functional theory (DFT) approach [24] and investigation of its antiviral activity using molecular docking [14].

To summarize, to the best of the authors’ knowledge, the study of possible structural states in solution by theoretical methods has not yet been carried out. The investigation on how these states manifest itself in spectra is important in order to develop methods for detecting and proper characterization of this substance, especially in cases of multiple routine or during flow measurements.

Optical spectroscopy methods such as e.g., vibrational spectroscopy and UV-visible-NIR absorption spectroscopy could be promising techniques for metabolites investigation. Their advantages include high accuracy and relatively good sensitivity to sample structure, as well as feasibility. Another advantage of this technique is the ability of spectral properties modelling [25] using advanced quantum chemical approaches, e.g., DFT. These methods possess significant predictive power and are computationally cost-effective. Theoretical studies conducted using this method contribute to the accumulation of knowledge about the object under study, allowing for more targeted experiments, saving time, effort, and material resources. This can be especially important in conditions of limited resources. The obtained theoretical knowledge can be compared with experiments to substantiate the obtained results and the chosen approaches.

Therefore, this study aims to theoretically investigate the structural features of the bikaverin molecule in solution and obtain possible low energy states. This research is also devoted to the characteristic spectral features inherent to identification of particular states, i.e., the structure–spectrum correlation when modeling vibrational properties and electronic transitions in the UV-vis region. Chloroform, a practically important solvent, was selected for the modelling medium. The obtained results have potential for use in developing methods for detecting the presence of this substance as a fungal metabolite, as well as for structure and quality control during the synthesis of medicinal substances containing it.

## 2. Results and Discussion

### 2.1. Structural Peculiarities

Since the bikaverin molecule shares structurally similar hydrogen bonds and functional groups with other previously studied pigments of the *Fusarium* genus, such as bostricoidin [25], rubrofusarin [26] and anhydrofusarubin [27], this study explored possible stable rotational conformers and tautomers to identify potential low-energy states. However, this article does not consider equivalent conformers in which the molecule transforms into itself. Such a transformation takes place around the C_Me_-O and C_Me_-C_ar_ bonds.

It was found that the stable structural states have C_s_ symmetry. Their structural peculiarities are demonstrated in Table 1. The optimized geometries of the states are listed in Section S1 in Supporting Information.

#### 2.1.1. The Lowest Energy State

As far as the authors know, there is only one study devoted to the investigation of crystal structure with bikaverin molecule [1]. The study [1] provides the bond lengths and bond angles data for the case of chloroform solvate of bikaverin in crystalline state. In this study, the results from [1] were compared with performed DFT calculations.

**Figure 1:**
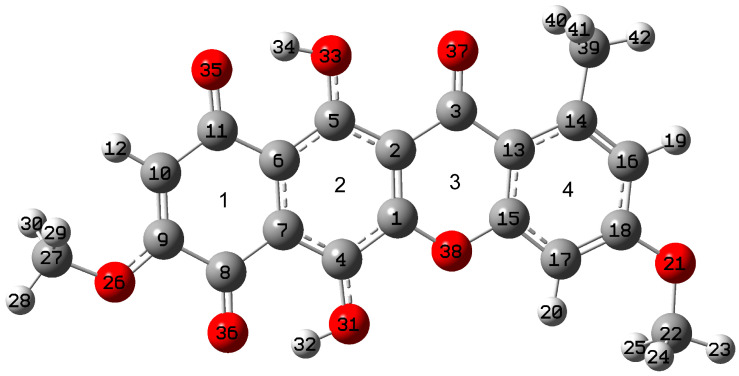
Image of optimized structure of bikaverin molecule in lowest energy state 1 with rings numbering. Here and after the light grey, dark grey, and red colors are used for hydrogen, carbon, and oxygen atoms in molecule.

The DFT calculations were performed at the B3LYP/6-311G(2d,p) level for the case of the chloroform solution. The solvent effect was taken into account implicitly within the polarizable continuum model. An image of the optimized structure of the lowest energy state with atoms and ring numbering is shown in Figure 1. Further in this study, this state will be called simply state 1. In agreement with [1], state 1 has C_s_ symmetry and flat polycyclic skeleton. For the lowest energy state 1, it was found that the dihedral angle C16C18O21C22 is 180°. Meanwhile, in case of crystal of chloroform solvate of bikaverin, this is 0°. It can be explained by the presence of intermolecular interactions in the crystal which was not taken into account in the single molecule calculations. Moreover, this fact reflects the possible rotations along the C18O21 bond in single molecule, which will be discussed further in Section 2.1.2.

Comparing the experimental and theoretical bond lengths of state 1, it can be concluded that mostly difference is within 0.025 Å. This is comparable with the standard deviation for experimental values 0.02 Å, as pointed out in [1]. The main differences occur for the two types of bonds. The first type is O26C27, C5O33, O38C1, C14C39, C18O21 and O21C22 (Table 1). It is the non-hydrogen bonds of functional groups (Me and MeO) attached to the polycyclic skeleton. Mostly it is C-O bonds. We suppose that the difference occurs due to the intramolecular interaction as well as underestimation of long-range interaction. The latter may lead to the bond lengths being predicted to be shorter than in the experiment. Another type is the bonds localized in the 4th ring, namely C13C14, C14C16 and C16C18.

Qualitatively the relations between the various calculated bond lengths in bikaverin molecule in state 1 are the same as in the experimental ones. According to Table 1, the shortest non-hydrogen bonds are C=O, i.e., C3O37 < C8O36 < C11O35. The bond length relation C8O36 < C11O35 correlates with O31H32 < O33H34 and H34O35 < H32O36 simultaneously. These suppose that the hydrogen bond O33-H34---O35 is stronger than the O31-H32---O36 in state 1.

Various lengths of double carbon bonds in hydroquinone (ring 2) and benzene rings (ring 4) as well as C9C10 in quinone ring are predicted to be within 1.353–1.435 Å. The C-C bond lengths within polycyclic part is predicted to be within 1.454–1.492 Å, while the C14C39 is 1.507 Å. The C-O bond lengths in ring 3 is 1.368 and 1.345 Å for C15O38 and O38C1 correspondingly. In case of hydroxyl groups, it is 1.333 and 1.325 Å for C4O31 and C5O33 correspondingly. In case of MeO-functional groups the C_Ring_-O and O-C_Me_ bond lengths are 1.349–1.331 and 1.429–1.433 Å.

In the naphthazarin part, the greatest difference in flat angle value between theory and experiment occurs with C6C11O35 (about 3.89°). Significant differences are also present with several C11 atom containing angles C10C11C6, C11C6C7 and C10C11O35. This may be due to the fact that it is the C11=O35 bond, which is involved in the formation of a stronger hydrogen bond, that is quite sensitive to the environment. Also, some differences were noticed for angles C4C7C8 and C5C6C11. In case of ring 3, a similar situation occurs with C3C2C1 and C2C3O37 angles. In case of ring 4, due to the different orientation of MeO- group and different interaction with environment the corresponding angles (C13C14C16, C14C16C18, C16C18O21, C18C17C15), values are also significantly differentiated from the experimental data in [1] (Table S1). Other theoretically predicted nonhydrogen angles coincide with experimental data within 1°.

#### 2.1.2. Conformational Isomers of Bikaverin

In the bikaverin molecule, conformational isomers (rotational conformers) can be formed by rotation around the C_Ar_-O single bond, which is present at the interface between polycyclic backbone and the attached MeO- and hydroxyl groups. One of these methyl groups is attached to the quinoid ring (ring 1), and another one is attached to the opposite side—to the benzene ring (ring 4) of the polycyclic backbone.

**Figure 2:**
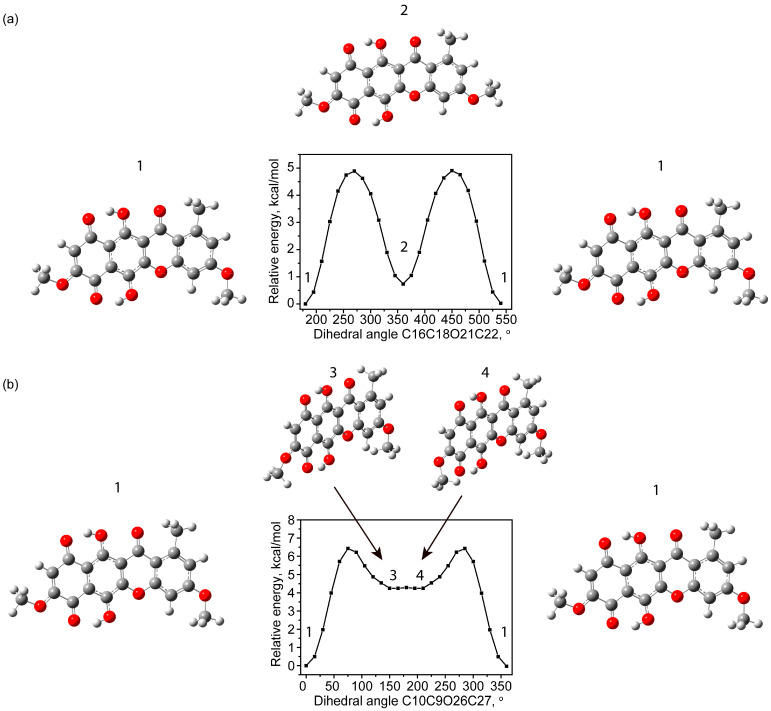
Scan of total potential energy with a change in dihedral angle C16C18O21C22 (**a**) and C10C9O26C27 (**b**).

The total potential energy scan for changes in the dihedral angle C16C18O21C22 on the benzene ring side is shown in Figure 2a. The most stable state corresponds to a value of C16C18O21C22 of 180°. At the same time, the scan revealed the presence of another stable state with a total potential energy slightly greater by 0.74 kcal/mol and C16C18O21C22 of 0°. The corresponding state was called state 2. This structure is closer to the observed experimentally in [1]. The most significant difference in the bond lengths between the state 1 and state 2 is observed in ring 4. Elongation of the C15C13, C14C16 and C18C17 bonds is observed with simultaneous shortening of the C13C14, C16C18 and C17C15 bonds (Table 1). The C14C16 bond is elongated the most (+0.008 Å), while the C13C14 (−0.007 Å) and C17C15 (−0.008 Å) bonds become the shortest. When comparing the lengths of hydrogen bonds and contacts for two states (1 and 2), a small (+0.003) increase in the length of the H32---O36 bond can be identified, which indicates its weakening. Among the flat angles, the greatest differences between two states occur in ring 4 with the angle C16C18O21. It becomes equal to 124.37 degrees. For comparison, in the experiment it was 125.4 degrees. However, it should be noted that, in general, when comparing with experimental angles in ring 4, greater differences are observed than for angles in other rings (see Table 1).

The proximity of energies may be due to the tendency of two hydrogens of the methyl group to form the longest contacts with the nearest hydrogen of the benzene ring. In this case, the remaining hydrogen is oriented towards the most distant hydrogen of this benzene ring. Small differences in energy may occur due to the different environments of hydrogens H19 and H20. The transition from one conformational state to another occurs with a rotation of 60° around the C_Me_-O bond. In this case, the potential barrier is 4.9 kcal/mol when estimated within the framework of this theoretical approach. Close values of potential barrier energies (4.54 kcal/mol in methanol solution) as well as differences in the energies of conformers (0.82 kcal/mol in methanol solution) were noted for the case of rotation around the C_Ar_-O bond when modeling by the same method, but in a different solvent for similar rotation in the rubrofusarin molecule [26].

Another scan of the total potential energy was carried out with the participation of the –OMe functional group located near the quinoid ring (Figure 2b). With this approach, the dihedral angle C10C9O26C27 was changed. The most stable state 1 corresponded to a value of 0°. In this case, states with an energy increased by 3.11 kcal/mol are noted, corresponding to the formation of H29---O36 (state 4) and H30---O36 (state 3) contacts. States 3 and 4 are enantiomers with respect to the mirror plane that passes through the polycyclic skeleton. Conformers 3 and 4 have a relatively low potential barrier between themselves (about 0.035 kcal/mol). At the same time, for the transition from state 1 to state 3 (or 4), it is necessary to overcome a potential barrier of about 6.42 kcal/mol. The relatively high potential barrier and potential electron energy make these states sparsely populated, and therefore of little interest.

Qualitatively, the observed features for this total potential energy scan are consistent with previously observed features for anhydrofusarubin [27].

**Figure 3:**
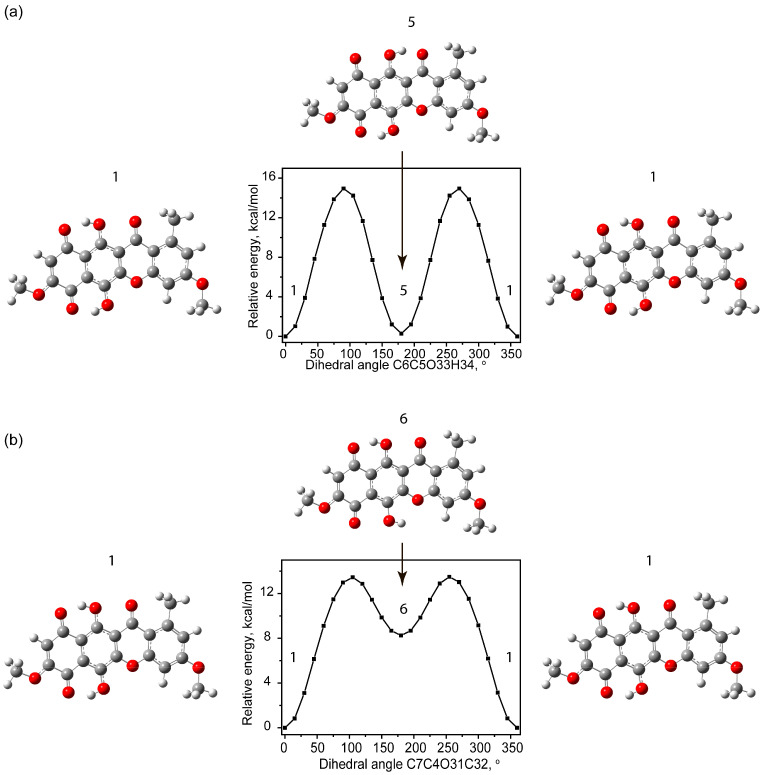
Scan of potential energy with a change in dihedral angles of C6C5O33H34 (**a**) and C7C4O31C32 (**b**).

Another type of rotational conformers occurs due to the rotation of the hydroxyl groups O33H34 and O31H32 relative to the carbon–hydrogen bonds. A characteristic feature of such rotations is the bending of the polycyclic backbone and carbonyl groups near the rotating OH group. This bending is associated, on the one hand, with increased repulsion of the electronegative oxygens, and on the other, with the formation of a hydrogen bond as the distance between the hydrogen of the hydroxyl group and the acceptor oxygen of the carbonyl group decreases. All this requires significant energy expenditure, which leads to high potential barriers for this type of transition. Figure 3a,b show potential energy scans for changing the dihedral angles of C6C5O33H34 and C7C4O31C32.

For the case of rotation of the O33H34 group (conventionally called the upper case), the potential barrier was approximately 15 kcal/mol. Meanwhile, in state 5, the total potential energy was only 0.32 kcal/mol higher than the most stable state. The geometry, as in the case of states 1 and 2, is planar with C_s_ group symmetry.

If one compares state 5 with state 1, changes in the bonds for rings 1–3 can be noted. However, for state 5 such changes for ring 4 are relatively small compared to state 1. As a result of the repulsion of the carbonyl group C11O35, a deformation of ring 1 is noted for state 5. It is manifested in the simultaneous lengthening of the bonds C6C11 and C11C10, as well as C7C8 and C8C9. At the same time, the angle C10C11C6 decreases slightly (−1.2°), while the angle C7C8C9 increases slightly (+0.6°). The most significant repulsion is manifested for the pair of angles C6C11O35 (+1.94°) and C10C11O35 (−0.82°). Also, for state 5, a reduced length of the C11O35 bond (1.226 Å) is noted (Table 1). For ring 2, the most significant differences are observed on the O31H32 hydroxyl side and at the boundary with ring 1. Specifically, the C4C7 and C7C6 bonds are lengthened, while the C1C4 and C6C5 bonds are shortened. This is also reflected in the corresponding angles: increased values for C5C6C11 (+1.92°) and C6C5O33 (+1.11°), and decreased values for the C4C7C8 (−1.4°) and C5C6C7 (−1.31°). Moreover, state 5 exhibits longer C-O bonds compared to state 1.

At the same time, ring 3 exhibits the formation of a hydrogen bond through the oxygen of the C3O37 carbonyl group. This is also manifested in structural aspects, for example, the reduced (−1.63°) value of the angle C2C3O37 with the increased (+1.27°) value of the angle C2C3C13 and (+2.7°) O38C1C4.

Also, for state 5, it is worth noting the smaller values for the O33H34 bond length and the increased values of the O37H40, O37H41 bond lengths (+0.012 Å). The length of H34O37 is greater than that of H34O35 in state 1. This indicates a lower strength of the hydrogen bond in O37---H34O33. The distortions of the structure of rings 1 and 2 also correlate with a decrease in the length of H32O36 (−0.032 Å).

For the hydroxyl group O31H32 (conventionally called the lower case), state 6 exhibits an increased C4C7C8 angle of approximately 2°, while the O38C1C4 angle decreases. Moreover, the energy of state 6 (+8.29 kcal/mol) is very high compared to the ground state, making it hypothetically sparsely populated. The potential barrier is approximately 13.49 kcal/mol.

Thus, the study of the energy states revealed the presence of three rotational conformers with comparable energies: states 1, 2 and 5. These conformers will be used below to model their optical properties.

#### 2.1.3. Tautomerism

The phenomenon of tautomerism in bikaverin was predicted in [19] based on comparison with other naphthoquinones with a similar structure, such as naphthazarin. However, to the authors’ knowledge, there are currently no studies on the theoretical study of tautomerism processes.

In this study, the tautomeric states were searched for via scanning the total potential energy as the hydrogen bond distance between the hydrogen of the hydroxyl group and the oxygen of the adjacent carbonyl group (=O---H) varied.

**Figure 4:**
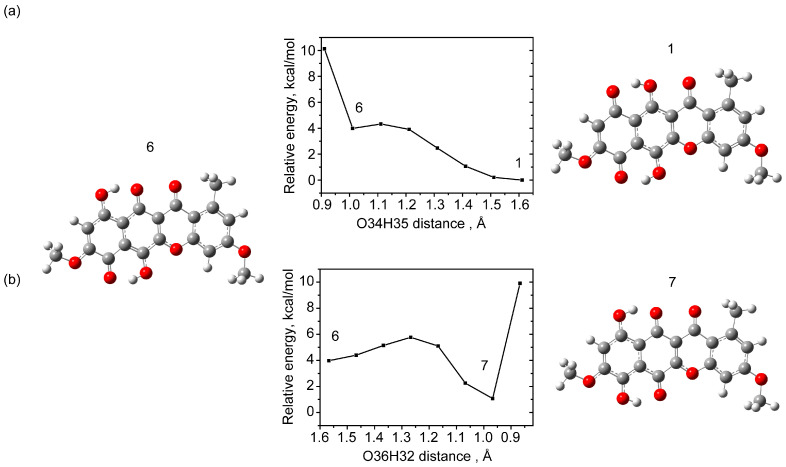
Example of formation of tautomeric states resulting from transfer of a hydrogen atom from a hydroxyl group: potential energy scan with O34H35 distance change (**a**), potential energy scan with O36H32 distance change (**b**), with respect to total potential energy of state 1.

The lowest-energy tautomeric state corresponded to the geometry where the hydroxyl groups O35H34 and O36H32 are formed—state 7 (Figure 4). The total energy of this state is approximately 1 kcal/mol higher than that of the ground state. For comparison, calculations for the anhydrofusarubin molecule in the gas phase [27] predict tautomeric energies 0.78 kcal/mol higher than the most stable state found.

When scanning with varying distances between H34O37 atoms, only an increase in the total potential energy was observed as the distance decreased; no stable tautomeric state was detected.

### 2.2. Vibrational Spectroscopy Analysis

#### 2.2.1. IR Absorbance Spectrum of State 1

**Figure 5:**
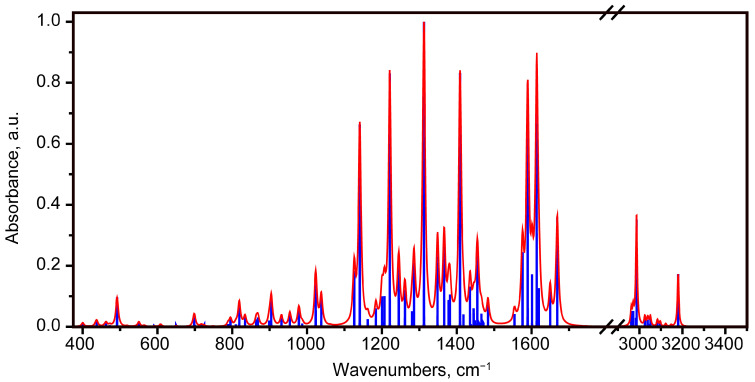
Theoretical IR absorption spectrum with scaled frequencies for molecule corresponding to state 1.

The calculated IR spectrum for the most stable state 1 is shown in Figure 5 presented in the practically important 375–3500 cm^−1^ region of the mid-IR as well as in supporting information in Table S2.1. Its lower boundary determines the absorption in the potassium bromide beam splitter of Fourier transform IR spectrometers, as well as when used potassium bromide in transmission experiments as a matrix for sample tablets preparation.

In the calculated IR absorption spectrum, several spectral ranges can be distinguished where vibrational modes appear.

The highest frequency range is associated with stretching vibrations in CH and OH bonds, with predominant atomic displacements of hydrogen atoms. The frequencies of these vibrations are usually overestimated due to the anharmonicity of the X-H vibration [28]. Moreover, according to [29,30], C-H stretching vibrational modes have specific frequencies depending on the hybridization of the carbon containing the hydrogen. Based on the relationships and proximity of frequencies with certain types of C-H stretching vibrations, this allows for the approximate localization of the O-H stretching vibrations frequencies, which, due to the formation of hydrogen bonds, can have quite variable frequency positions.

Thus, the experimentally determined ranges for symmetric and antisymmetric vibrations in methyl groups are 2870 ± 10 cm^−1^ and 2960 ± 10 cm^−1^, respectively. In the calculation, the symmetric stretching vibrations correspond to the scaled frequency range of 2962–2984 cm^−1^ (modes No. 107-109). Mode No. 110 corresponds to the calculated scaled frequency of 2987 cm^−1^, related to vibrations in the hydroxyl group O33H34. It is located at the high-frequency boundary of the symmetric vibrations range in the methyl group, which allows to assume its experimental position at about 2880 cm^−1^. Due to the C_s_ symmetry the antisymmetric modes of the methyl groups in the molecule are divided into antisymmetric water-like vibrations (modes 111–113) and antisymmetric stretching vibrations of CH_3_ with much larger atomic displacements in the mirror plane (modes 114–116). Modes 117–119 correspond to stretching hydrogen vibrations in the polycyclic part (ν(=C-H)). Their characteristic frequency range is 3000–3100 cm^−1^. The scaled stretching frequency of the O31H32 bond (mode 119) is predicted by calculation to be near the high-frequency limit for ν(=C-H); thus, in the experiment it should be expected to be around 3100 cm^−1^.

A qualitatively similar ratio of vibrational frequencies in the OH groups was observed for anhydrofusarubin. The stretching vibration closer to the MeO group attached to the naphthazarin moiety had a higher frequency. However, the predicted vibrational frequencies were higher, which is partly due to the fact that this pigment was calculated in the gas phase.

The most active ones in the IR absorption spectrum of bikaverin are predicted to be the OH stretching vibrations. However, it should be highlighted that the hydrogen stretching vibrations are sensitive to the environment and their bands can be broadened and/or shifted in experimental conditions.

In the region below 1700 cm^−1^, calculations reveal a distinct set of localized peaks. In the scaled spectrum, this spectral range includes modes No. 99-106 located in the range 1525–1700 cm^−1^. A characteristic feature of these modes is that they are located in the spectral frequency range characteristic of δ(COH) bending vibrations, as well as ν(C=C) and ν(C=O) stretching vibrations. This leads to delocalization of atomic displacements in these bonds.

**Figure 6:**
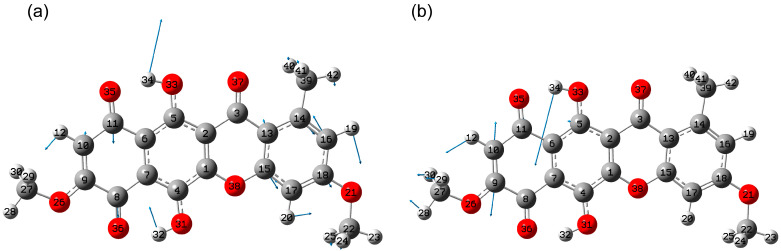
Amplitudes of atomic displacements in modes 103 (**a**) and 101 (**b**).

In this case, the different strength of the C_Ar_-OH---O=C type intramolecular hydrogen bond is influenced by the solvent or medium and the environment of the highly polar C=O bond in particular. These can lead to shifts in the frequencies of these vibrations. In [21], a set of IR absorption peaks is given for bikaverin 1665, 1643, 1614 and 1589 cm^−1^. In [22] peaks are noted at 1668, 1616, 1584 and 1564 cm^−1^, in [19] 1670, 1653 and 1620 cm^−1^, and in [20] 1650 and 1610 cm^−1^. The highest activity in the IR spectrum is predicted for modes 103 and 101 with scaled frequencies of 1614 and 1590 cm^−1^ (Figure 6). In mode 103, delocalized vibrations in the C=O bonds in the quinone ring (1) are observed, as well as δ(COH) together with the C=C stretching vibrations in the benzene (4) and quinone (1) rings. It corresponds to the experimental peak observed in various studies [19,20,21,22] in the range of 1610–1620 cm^−1^. In mode 101, the atomic displacements are predicted to be much more localized and correspond to vibrations mainly in the naphthazarin part, the largest atomic displacements are δ(C5O33H34), δ(C10C9) and δ(H12C10C9). It corresponds to the experimentally observed peak in the range 1584–1589 cm^−1^ [21,22]. Modes 106 (1669 cm^−1^) and 105 (1650 cm^−1^) appear weaker, but located separately. Mode 106 corresponds to the observed peak 1665–1670 cm^−1^ in the [19,21,22]. It is interpreted as predominantly ν(C3O37) vibrations. Mode 105 corresponds to the peak in the range 1610–1620 cm^−1^ observed in some articles [19,20,21,22]. It is interpreted as stretching vibrations in the ν(C8O36), ν(C3O37) and δ(C4O31H32) bonds, as well as numerous carbon–carbon stretching vibrations with atomic displacements of the benzene ring mode v8a in rings 1 and 2.

It should be noted that modes 97 and 98 are also characterized by the presence of a contribution from ν(C=C) and δ(COH), together with δ_asym_(CH_3_), but the activity of these modes is relatively weak.

Modes 89–96 (range 1438–1471 cm^−1^) are characterized by, among other things, antisymmetric bending atomic displacements in the methyl groups. The most active in this range is mode 92 (1455 cm^−1^), its atomic displacements are localized in the region of ring 4 and include antisymmetric bending vibrations in C39H3 and C22H3, as well as carbon–carbon stretching vibrations in the 4th (benzene) ring, similar to mode v19a in Wilson notations for benzene ring. Hereafter, the vibrational modes starting with the “v” refer to the normal modes in benzene rings in Wilson notations.

Modes 81–88 (1348–1435 cm^−1^ range) are characterized by the presence of water-like atomic displacements in the polycyclic core. These vibrations are coupled with hydrogen bending motions in methyl groups, CCH, and COH moieties, as well as stretching vibrations in single bonds. The relative contribution of polar vibrations, especially δ(COH) and ν(CO), and their phase determine the mode’s activity in the IR spectrum.

The most active vibrational mode in this range is 86 (1409 cm^−1^). It has high polarity, which is associated mainly with δ(C5O33H34), ν(C5O33) and δ(C4O31H32), ν(C4O31), as well as atomic displacements of carbons in the 2nd ring like the v19a mode of the benzene ring and v_asym_(C10C11C6). For mode 81, the carbon displacements are localized in the 4th ring and are close to the v19b mode in benzene, as well as in the polar parts of the molecule δ(C4O31H32), ν(C18O21) and ν(C15O38). For mode 82, the atomic displacements are localized mainly in the region of the MeO-naphthazarine part. In ring 1, the atomic displacements of carbons are close to those in the v19b mode in benzene. In addition, significant atomic displacements occur in δ(C5O33H34), δ(C6C5) and δ(C9C10H12).

Mode 80 (1312 cm^−1^) exhibits relatively high activity in the IR absorption spectrum. It corresponds to hydrogen deformation vibrations δ(C5O33H34), δ(C4O31H32) and δ(C9C10H12), conjugated with stretching vibrations in single bonds, the largest shift in which is ν(C8C9), ν(C7C8), ν(C2C3) and ν(C5O33), which lead to distortion of the shape of the 1st and 2nd rings.

**Figure 7:**
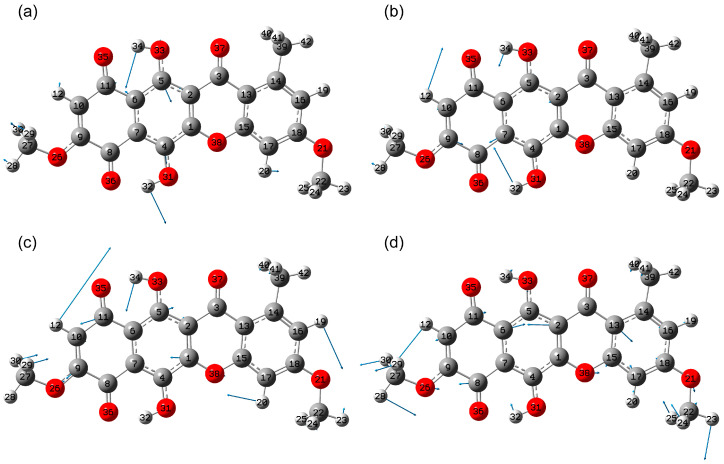
Atomic displacements in vibrational modes 86 (**a**), 80 (**b**), 75 (**c**) and 68 (**d**).

In the region of 1100–1300 cm^−1^, the calculation predicts two most active modes: No. 68 (1141 cm^−1^) and 75 (1221 cm^−1^) (Figure 7). Atomic displacements in mode 75 are quite strongly delocalized. The largest stretching vibrations occur in a number of single bonds, including O26C9, C10C11 and C1O38. A contribution from stretching deformation vibrations is also noted, mainly δ(C9C10H12), δ(H19C16C18), δ(C5O33H34) and δ(C15C17H20), as well as wagging type motions in H29C27H30. For the accompanying mode 68 (1126 cm^−1^), the pattern of atomic displacements is qualitatively similar (Figure 7b). The main atomic displacements are as follows: δ(C15C17H20), v(C1O38), δ(C8C7C4), δ(H19C16C18), δ(H32O31C4).

In turn, mode 68 in the polycyclic skeleton is characterized by a combination of stretching vibrations in single bonds in rings 1 and 3 (C10C11, C2C3, C3C10, O38C15, C18O21), as well as deformation vibrations in structural units with double bonds (O26C9C8, C6C5C2, C15C13C14) (Figure 7d). In addition, ρ(C27H3) and ρ(C22H3) are noted.

For less active modes 77–79 (1261–1286 cm^−1^) a shifted character of stretching atomic displacements in double bonds (rings 2 and/or 4, the type is close to the v19b mode) with stretching vibrations in single bonds (in rings 1 and/or 3 and at the boundary of the polycyclic backbone of the molecule) is characteristic. For other less intense modes, No. 76 (1162 cm^−1^), 74 (1207 cm^−1^), 73 (1200 cm^−1^) and 72 (1184 cm^−1^), three main contributions with different ratios are noted, associated with stretching vibrations in single bonds C-C and C-O, deformation vibrations of the δ(CCH) type and rocking modes in CH_3_ groups.

Modes 59–61 and 63–64 are characterized by a combination of stretching vibrations in single C-C and C-O bonds, as well as bending vibrations in the polycyclic skeleton. Thus, mode 63 is characterized by predominantly stretching vibrations in the C27O26, C2C36, and C4O31 bonds, as well as deformation vibrations in the O26C9C8, C7C4C1, C7C6C5, and C1C14C16 bonds. Atomic displacements are highly delocalized across all four rings. In contrast, in mode 64, atomic displacements are localized in the third and fourth rings; stretching vibrations are observed in the C1O38, O38C15, O21C22 and C14C39 bonds, as well as bending vibrations in ring 4 δ(C17C18C16) and δ(C18C16C14).

In the range of 375–904 cm^−1^, the calculation predicts modes that can be divided into two groups based on the type of atomic displacements. The first group includes modes that feature vibrations in the plane of the polycyclic core. The main contributions to these modes come from bending vibrations with changes in the bond angle in the core and at the interface with the CH_3_ and –O-CH_3_ groups.

**Figure 8:**
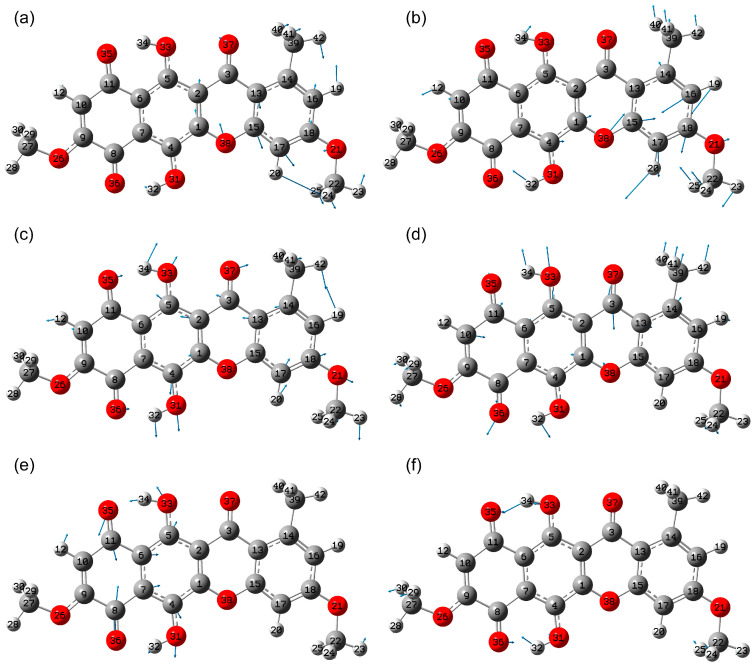
Amplitudes of atomic displacements in modes 44 (**a**), 37 (**b**), 34 (**c**), 32 (**d**), 30 (**e**) and 27 (**f**).

Among the most active modes, mode 51 (795 cm^−1^) stands out, in which the bond angles of C27O26C9, C7C4O31 and C4O31H32, as well as C10C11O35, C11C6C5 and C8C7C4 change. Rings 1 and 2 twist in opposite directions. For mode 44 (698 cm^−1^), atomic displacements are localized in the third and fourth rings and the MeO group attached to the last one, namely δ(C17C18O21), δ(C1O38C15), δ(O37C3C13), δ(C39C14C16), δ(C3C13C14), δ(C17C15O38). Ring 4 twists counterclockwise (Figure 8a).

Mode 37 (550 cm^−1^) is characterized by presumably compression–extension of ring 4 with changes in the C13C15C17 and C14C16C18 bond angles, as well as C18O21C22 (Figure 8b). This also results in slight deformations of rings 1–3, with changes primarily in the C1C15O38 and C1C2C3 angles.

For mode 34 (492 cm^−1^), calculations predict relatively high activity in this region of the IR absorption spectrum (Figure 8c). This vibration is characterized by in-phase atomic displacements due to changes in the C10C11O35, C9C8O36, and C2C3O37 bond angles. Furthermore, deformations of ring 4 occur due to changes in the C17C18C16 and C5C13C14 angles, as well as the attached methyl group C16C14C39. All this leads to a nearly horizontal orientation of the dipole moment derivative vector along the normal coordinate in the figure plane. In-phase changes in the C7C4C1 and C6C5C2 angles for ring 2 are also observed, leading to its compression–extension.

An out-of-phase compression–extension of ring 3 occurs for mode 32 (462 cm^−1^), relative to the compression–extension of the other rings (Figure 8d). The largest atomic displacements occur for the C9C10C11, C2C3C13, C27O26C9, O36C8C7 and C15C13C14 bond angles.

For mode 30 (438 cm^−1^), antiphase compression and extension of rings 1 and 2 occurs, with changes in the C10C11C6 and C9C8C7 angles, as well as antiphase changes in the C6C2C2 and C7C4C1 angles. Counterrotations of the =O35---H34-O33- and =O36---H32-O31- hydrogen bonds occur (Figure 8e).

Mode 27 (400 cm^−1^) is characterized by a change in angles involving atoms forming hydrogen bonds: C6C11O35 and C6C5O33, as well as C7C8O36 and C7C4O31. In this case, movements of oxygens (O33, O31) and hydrogens (H34, H32) towards oxygens (O35, O36) are observed (Figure 8f). The manifestation of the strength of the hydrogen bond here lies in the ratio of the amplitudes of atomic displacements for hydrogens H34 and H32. The stronger the hydrogen bond, the greater the amplitude of hydrogen atomic displacement in this vibrational mode.

The second group includes modes with out-of-plane atomic displacements. Among them, the vibrational modes 53 (819 cm^−1^), 54 (834 cm^−1^), 55 (864 cm^−1^), 56 (869 cm^−1^) and 58 (904 cm^−1^) have the highest activity in the IR absorption spectrum. Modes 53 and 58 are related to vibrations of hydrogen atoms forming an intramolecular bond of the type -O-H---O=, respectively, tors(C7C4O31H32) and tors(C6C5O33H34). The greater strength of the O33H34---O35 hydrogen bond compared to O31H32---O36, estimated from the stretching vibration frequencies given above (the frequency ν(O33H34) is lower than that of ν(O31H32)), leads to the fact that the torsional vibration frequency for mode 58 is higher than that of mode 53.

A characteristic feature of out-of-plane modes 54, 55 and 56 is that they are located near double carbon bonds, namely tors(C13C15C17H20), tors(C17C18C16H19) and tors(C6C11C10H12).

To sum up, in this section, the IR absorption spectrum for the most stable state of the bikaverin molecule was interpreted and compared with experimental data available in the literature. As can be seen, for various vibrational modes—bond stretching, bond bending and out-of-plane (torsional) vibrations—a number of modes with significant atomic displacements of hydrogen atoms, forming intramolecular hydrogen bonds, are predicted.

#### 2.2.2. IR Absorbance Spectra Peculiarities of States 2, 5 and 7

This section examines spectral features typical of states 2, 5, and 7, which can be used to identify these states using IR absorption spectra in various ranges. The goal of this section is to identify reliable highly active peaks or bands consisting of several peaks separated from other maxima by >10 cm^−1^ in frequency (this range is taken into account based on the spectral resolution of the instruments, as well as possible broadening caused by the environment). These bands can be used as markers of a specific structural state, taking into account the possible overlap of individual vibrational mode contours. The interpretation presented in Section 2.2.1 is used to justify the selection of these bands. The listing of the calculated vibrational mode frequencies and their activities in IR absorbance spectrum is present in Tables S2.2–S2.4 for states 2, 5 and 7, correspondingly, in supporting information.

##### Hydrogen Bond Stretching Region

**Figure 9:**
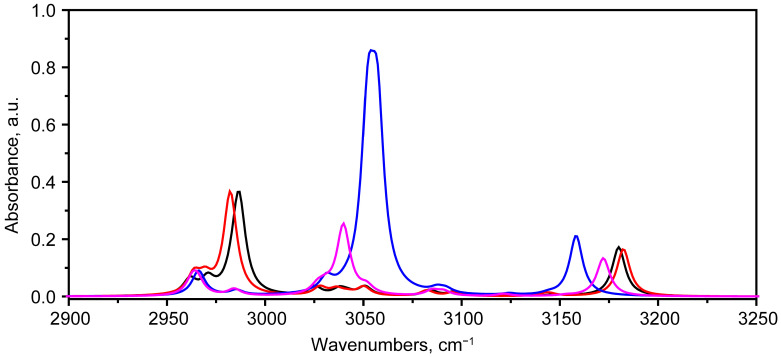
Scaled IR absorbance spectra corresponding to bikaverin molecules in states 1 (black), 2 (red), 5 (blue) and 7 (purple), calculated at B3LYP/6-311G(2d,p) level in chloroform in region of 2950–3250 cm^−1^.

Figure 9 shows a portion of the IR absorption spectrum corresponding to the region of hydrogen stretching vibrations.

As can be seen from a comparison of the contours in this range for states 1 and 2, the calculation predicts relatively small differences within 5 cm^−1^. As noted in Section 2.1.1, state 2 exhibits differences in the geometry of ring 4 and the H41---O37 and H40---O37 hydrogen bonds. This also manifests itself in the length of the O35---H34 hydrogen bond, which is shorter by 0.001 Å in state 2. However, due to the high vibration frequency, this nevertheless leads to a decrease in frequency by about 5 cm^−1^. The opposite situation is true for the O36---H32 bond: the bond length increases slightly, leading to an insignificant increase in frequency.

The situation changes qualitatively for state 5. The close proximity of C39H3 to O37 and the corresponding hydrogen bonds H41---O37 and H40---O37 lead to the formation of a weaker hydrogen bond H34---O37 compared to H34---O35 in state 1 resulting in an increase in the stretching vibration frequency ν(H34O33) and coupling with ν_asym_(C39H3) in state 5. As a result, two vibrational modes 113 (3052 cm^−1^) and 114 (3057 cm^−1^) with a significant contribution of ν(H34O33) are predicted. They form a relatively intensive IR active band.

In the case of the tautomer (state 7), the environment different from state 1 also leads to a weakening of the bond, which was manifested in longer distances of H34O33 (1.613 Å) and H32O31 (1.667 Å) in state 7 compared to H34O35 (1.594 Å) and H32O36 (1.665 Å) in state 1. And, as a consequence, this led to an increase in the frequency of the 112 ν(O35H34) mode by 53 cm^−1^. At the same time, despite a slight increase in the bond length, a slight decrease in its frequency (−8 cm^−1^) was noted for the mode 120 ν(H36O32).

##### The Region of 1494–1700 cm^−1^

**Figure 10:**
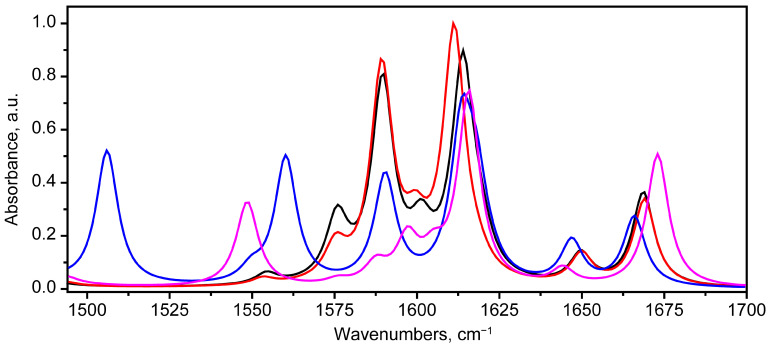
Scaled IR absorbance spectra corresponding to bikaverin molecules in states 1 (black), 2 (red), 5 (blue) and 7 (purple), calculated at B3LYP/6-311G(2d,p) level in chloroform in 1494–1700 cm^−1^ region.

The scaled IR absorbance spectra corresponding to the bikaverin molecules in states 1 (black), 2 (red), 5 (blue) and 7 (purple) in the 1494–1700 cm^−1^ region are presented in the Figure 10.

A comparison of the calculated scaled IR absorption spectrum contours for states 1 and 2 reveals their relative similarity. Minor frequency differences are noted for the 1614 cm^−1^ band (state 1) compared to 1611 cm^−1^ in state (2), which is due to the contribution of the vibrational mode from the stretching vibrations in ring 4, whose geometry undergoes relatively minor changes.

**Figure 11:**
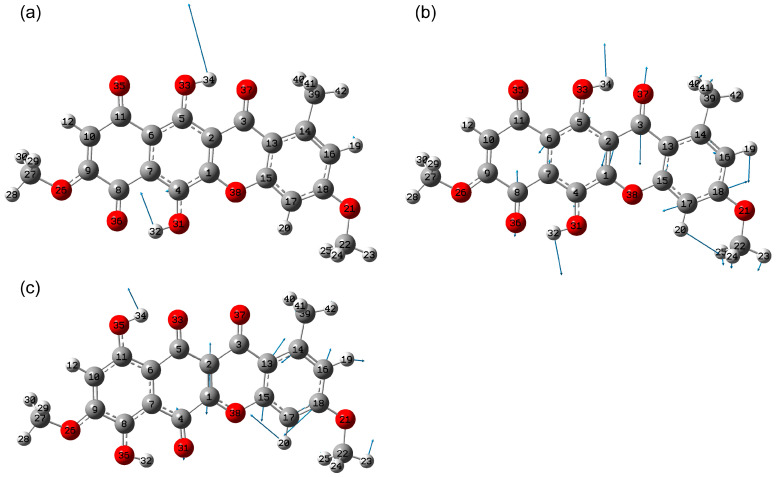
Atomic displacements in mode 98 (**a**) and 100 (**b**) for state 5 and 99 for state 7 (**c**).

Much more noticeable differences are observed for state 5. For this state, vibrational modes at 100 (1560 cm^−1^) and 98 (1506 cm^−1^) are predicted to appear in the spectrum (Figure 11a,b). A characteristic feature of these modes is the presence of significant atomic displacements δ(COH), including δ(C5O33H34) with a different environment.

For the tautomer (state 7), the calculated spectrum contour in the range 1494–1700 cm^−1^ differs significantly. A separated peak corresponding to vibrational mode 99 in state 7 appears (Figure 11c). In state 1, mode 100 (1575 cm^−1^) is closest to it in terms of the atomic displacements pattern. However, mode 99 in state 7 lacks the contribution from δ(C8O36H32), i.e., the second of the two deformation vibrations with an OH group, which may lead to a decrease in the frequency of the vibrational mode.

##### The Region of 1100–1494 cm^−1^

In this range, the peaks of vibrational modes from different states are quite close in frequency.

Because the influence of the environment, especially for light hydrogen, can lead to broadening and merging of the individual vibrational modes peaks, the presence of broad bands in the experimental spectrum cannot be ruled out.

Therefore, within this range, it is more appropriate to present and discuss vibrational modes that differ significantly from the lowest-energy state 1.

For the tautomer (state 7), vibrational modes with the following features are distinctive from state 1:

1. The presence of a significant contribution from deformation vibrations in δ(C11O35H34) and δ(C8O36H32);
2. Carbon–carbon stretching vibrations in the rings of hydroquinone, whose environment in state 1 differs qualitatively from state 7, where hydroquinone is located at the edge and an MeO group is attached to it.

**Figure 12:**
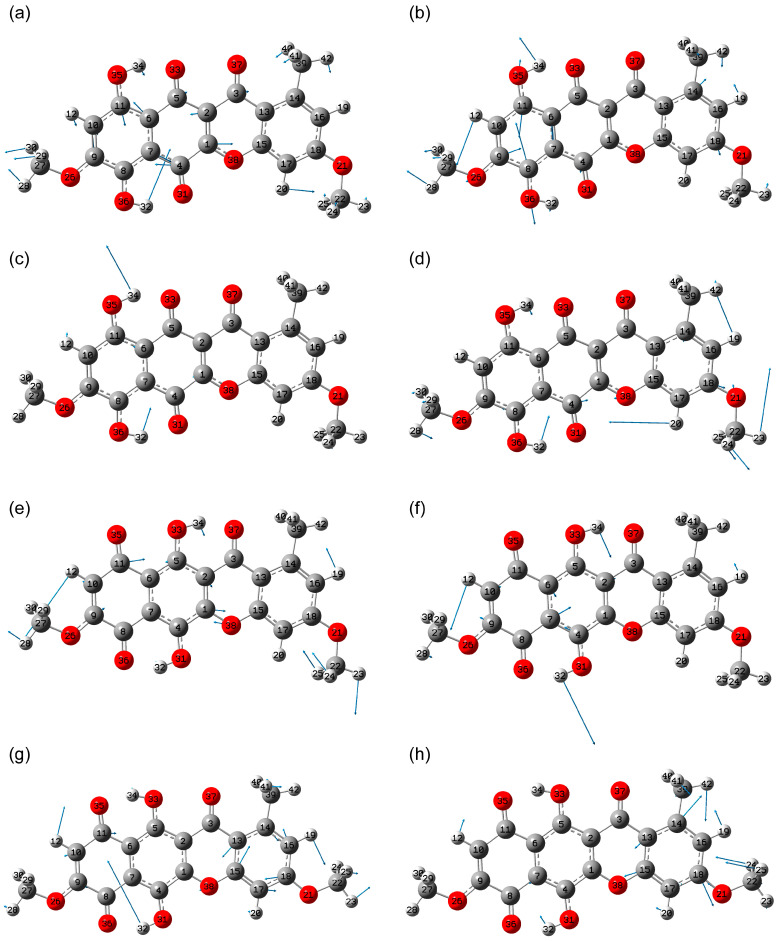
Atomic displacements in 85 (**a**), 79 (**b**), 81 (**c**), 71 (**d**) modes in state 7; 74 (**e**) and 80 (**f**) mode in state 5; 81 (**g**) and 79 (**h**) modes in state 2.

Specific vibrational modes that do not overlap with other vibrational modes in frequency include modes 85 (1395 cm^−1^) and 79 (1285 cm^−1^), with significant vibrations in the hydroquinone moiety, and 81 (1339 cm^−1^), with a significant contribution specifically from δ(C8O36H32) (Figure 12a–c). Mode 71 (1157 cm^−1^) can also be classified as specific, where the contribution from δ(C8O36H32) is weakly expressed. However, this mode overlaps with the frequencies of other vibrational modes (Figure 12d).

In the case of state 5, the most specific vibrational modes are 74 (1210 cm^−1^), with significant atomic displacements in the hydroquinone moiety and at its boundary, as well as mode 80 (1304 cm^−1^), which, in addition to ν(C=C) in the hydroquinone moiety, also features significant δ(C5O33H34) and δ(C4O31H32) vibrations (Figure 12e,f).

Considering the state 2, the most specific modes are 81 (1330 cm^−1^) and 79 (1301 cm^−1^) (Figure 12g,h). The latter, despite overlapping with the vibrational modes of states 5 and 7, still differs significantly in frequency from the closest mode 80 in state 1.

##### The Region of 375–1100 cm^−1^

**Figure 13:**
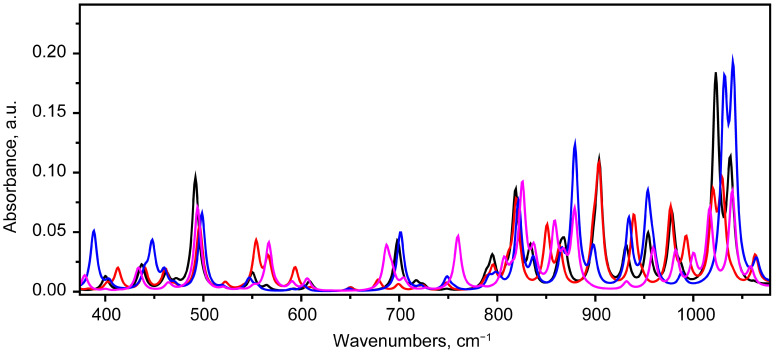
Scaled IR absorbance spectra corresponding to bikaverin molecules in states 1 (black), 2 (red), 5 (blue) and 7 (purple), calculated at B3LYP/6-311G(2d,p) level in chloroform in 375–1100 cm^−1^ region.

In the 890–1100 cm^−1^ range, significant frequency overlap is observed for vibrational modes of different states (Figure 13).

Therefore, a search for specific vibrational modes was conducted in the 375–890 cm^−1^ range. As noted previously in Section 2.2.1, this range contains vibrational modes that are either out-of-plane or have atomic displacements in the plane of the polycyclic backbone with ring deformation.

**Figure 14:**
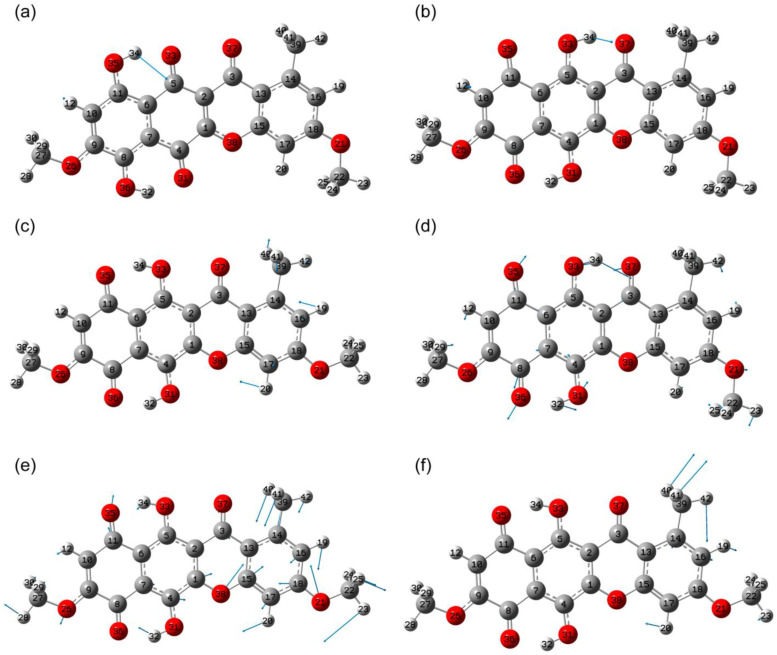
Atomic displacements in selected modes: 57 (**a**) in state 7, 56 (**b**) in state 5, 55 (**c**) in state 2, 30 (**d**) in state 5, 40 (**e**) and 62 (**f**) in state 2.

In this regard, the approach to searching for specific modes was as follows. As was shown earlier in Section 2.2.1, the frequencies of torsion modes with the hydrogen of the OH group depend on the strength of the intramolecular hydrogen bond, which in turn is determined by the –OH---O=C environment. In this regard, a comparison of vibrational frequencies was carried out for torsion modes of the tors(HOCC) type, which will be more specific for vibrations with the H34 hydrogen for states 5 and 7. Mode 57 (879 cm^−1^) and, to a lesser extent, mode 55 (858 cm^−1^) in state 7, surrounded by others, can serve as such a vibrational mode (Figure 14a,b). For state 5, mode 56 (879 cm^−1^) serves as a similar one.

For state 2, mode 55 (851 cm^−1^) is sensitive to the position of the MeO group, possessing the largest atomic displacements are in tors(C13C14C16H19) and tors(C16C18C17H20) (Figure 14c).

Another type of specific vibrational modes for different states may be modes with a planar type of atomic displacements, where deformation vibrations are present, for example, of the δ(CCO) type and/or stretching/bending/rotation of the hydroquinone part occurs.

Examples of such vibrational modes for state 7 (tautomer) include the following:

1. Mode 38 (567 cm^−1^), involving deformation of the hydroquinone moiety;
2. Mode 44 (689 cm^−1^), involving δ(CCO) and rotation of the benzene ring of the hydroquinone moiety;
3. Mode 49 (760 cm^−1^), involving δ(CCO) vibrations.

For state 5, modes 26 (388 cm^−1^) and 30 (448 cm^−1^) show significant contributions from δ(CCO) bending vibrations localized near hydrogen bonds.

Considering state 2, mode 40 (593 cm^−1^) shows deformations of ring 4 and fairly significant displacements for δ(C18O21C22), which determines the specificity of this mode. Also, deformations of the fourth ring are carried out in mode 62 (993 cm^−1^), where δ(C14C16C18) is also noted.

### 2.3. UV-Vis Absorption Spectroscopy

The UV-vis absorption spectroscopy has been frequently used to confirm the presence of bikaverin since early studies [8,22]. Experimental electron absorption spectra of bikaverin in chloroform show a broad long-wavelength band with a maximum near 507–510 nm [8,19]. In [19], a shoulder at approximately 550 nm was also noted. According to [22], a maximum at 520 nm for the longest-wavelength band of bikaverin in the given absorption spectrum in the 400–600 nm range, along with clearly distinguishable shoulders at 550–560 nm on the long-wavelength side and at approximately 490 nm on the short-wavelength side were observed. The contour of this band can be a consequence of several processes: the manifestation of an electron-vibrational fine structure, as well as the manifestation of stable states with different energies existing in the solution.

**Figure 15:**
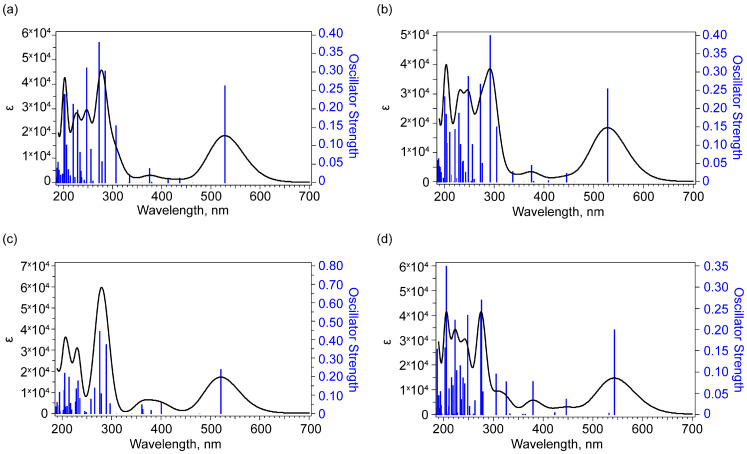
Calculated UV-vis absorbance spectra for vertical singlet-singlet transitions at B3LYP/6-311G(2d,p) level for case of molecule in chloroform for state 1 (**a**), 2 (**b**), 5 (**c**) and 7 (**d**).

Calculations in this region predict a transition to the first excited state for a single molecule (Figure 15, Table 2). For states 1 and 2, which differ in the position of the MeO group, the transition wavelength is approximately 528 nm. For state 5, the wavelength of the longest-wavelength transition is shorter—approximately 521 nm. And for the tautomer (state 7), the transition wavelength is near 543 nm.

Considering the 310–400 nm range, the experimental spectrum shows an increase in absorption as the short-wavelength boundary is approached. In [8], a shoulder near 336 nm in the spectrum was also noted. Calculations for states 1 and 2 in this region reveal a transition with the highest oscillator strength to the sixth excited state with a transition wavelength of approximately 375 nm. For state 5, theory also predicts a peak formed by transitions with the highest oscillator strengths in states 3 and 7, with corresponding transition wavelengths of 361 and 401 nm. In the case of tautomeric (seventh) state, the presence of transitions with comparable oscillator strengths to the 5th and 9th excited states with wavelengths of 380 and 326 nm, respectively, is noted. An alternative interpretation of the 336 nm shoulder is a transition with a wavelength of about 300 nm to the 10th excited state, which is noted for states 1, 2 and 7 (tautomer).

In the region below 300 nm, the experimental spectrum of bikaverin contains peaks at 253 and 271–276 nm [8,19], as well as a weaker peak at 228 nm [8]. In the predicted calculated spectra for states 1, 2, 6 and 7, the peak in the 271–276 nm range corresponds to two transitions with comparable oscillator strengths. For states 1, 2 and 5, the characteristic difference in wavelengths for these transitions is 11–13 nm, while for the tautomer (state 7) it is within 1.4 nm. The experimental peak at 253 nm corresponds to a calculated transition wavelength of approximately 248 nm. For states 1, 2 and 7, theory predicts a significant oscillator strength for this transition, in contrast to state 5. It should be noted that for state 1, the oscillator strengths for transitions forming a band with a maximum at approximately 273 nm are significantly greater than for the transition at approximately 248 nm. For states 2 and 7 (tautomer), the relative contribution from the 248 nm band is greater, which may indicate the existence of these states in solution.

In the 220–240 nm range, the band is formed by a set of transitions. Simulating the contour for states 1, 5 and 7, this band is distinct at a given half-width at half-maximum (0.19 eV = 1500 cm^−1^), while poor resolution is observed for state 5.

Around 200–205 nm, the calculated spectrum predicts a strong band for states 1, 2, 5 and 7. Due to the C_s_ symmetry of the molecule the most active transitions from ground to excited state have their transition electric dipole moment in polycyclic plane. 

**Figure 16:**
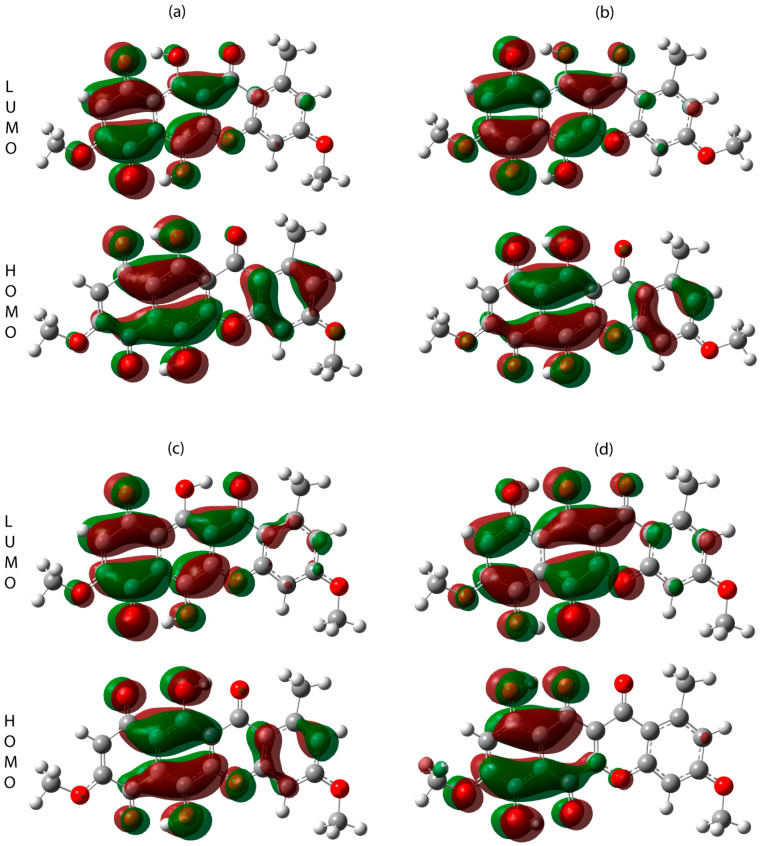
HOMO and LUMO orbitals for states 1 (**a**), 2 (**b**), 5 (**c**) and 7 (**d**).

Considering the found conformational states, their parameters associated with the energies of frontier orbitals were calculated (Figure 16, Table 3), i.e., ionization potential (I = −E_HOMO_), electron affinity (A = −E_LUMO_), electronegativity (χ = (I + A)/2), electrophilicity index (ψ = χ^2^/(2η)), chemical hardness (η = (I − A)/2) and chemical softness (S = 1/(2η)) [31,32]. The obtained results are demonstrated in Table 3. The ionization potential decreases in the row: state 1 > state 2 > state 5 > state 7. This means that the greatest energy is needed to remove the electron in case of state 1 and the lowest in case of state 7 (tautomer). State 5 demonstrates lowest electron affinity, HOMO-LUMO gap, electrophilicity and chemical softness. Therefore, it can be concluded that the state is more stable and less predisposed to receive electrons it is more difficult to perform charge transfer. The opposite situation is with tautomer (state 7), which has lowest chemical hardness and greatest chemical softness as well as electrophilicity.

## 3. Theoretical Approach

In the study, the calculations were performed within the density functional theory (DFT) approach using Gaussian G09W Rev. C.01 software [33]. It utilized the exchange correlation functional B3LYP [34,35,36] and the 6-311G(2d,p) basis set [37,38]. This approach has previously demonstrated good predictive ability in modeling structural, electronic, and vibrational properties [25,26,27,28,39,40]. Moreover, in case of some polycyclic molecules (e.g., alizarin), it was demonstrated that the B3LYP approach demonstrates fairly good results in predictive of electronic properties [41] which is typical for molecules with sufficient orbital overlap parameter [41,42]. The relaxed potential energy scans were performed with 15° step for rotational conformers and 0.1 Å for tautomers. Optimization was performed until the standard conditions for maximum and RMS values of displacements and forces were fulfilled. The stability of the determined geometries was checked during vibrational frequency calculations. It was found that imaginary modes were absent for the structural states 1, 2, 5 and 7 (see Tables S1.1–S1.4). In the text is discussed the scaled vibrational modes frequencies due to the fact that they are closer to experimental data. The scaling factor was equal to 0.98 on the basis of previous studies [25,27]. The calculated Raman activity and IR absorbance of the modes are demonstrated in Tables S2.1–S2.4. The effect of the solvent (chloroform) was implicitly taken into account within the polarizable medium model (PCM) with standard parameters. The PCM is based on the integral equation formalism. In such a model, the van der Waals surface type cavity is created which is made up of overlapping spheres. Such a cavity is created with the GePol algorithm [43] with standard parameters predefined in software including for sphere list used, Lebedev–Laikov grid of cavity, and polarization charges [33]. The standard dielectric permittivity (ε) for chloroform at 25 °C and 1 atmosphere was equal to 4.7113. In high frequency case the standard dielectric permittivity (ε^∞^) was equal to 2.090627 [33]. In case of optimization and further calculation of vibrational properties the calculations within PCM were performed in equilibrium way. On the other hand, in case of vertical transition energies the calculations within PCM were performed in non-equilibrium way. The determined geometries were further used for calculations of the 40 lowest vertical singlet-singlet transitions within the time-dependent DFT (TD-DFT). The electron absorption spectrum was simulated with a FWHM broadening of 0.19 eV (1500 cm^−1^) for each transition. It should be noted that due to the underestimation of the long-range interaction, such theoretical approach may predict the longer wavelength (smaller energies) transitions, in particular, the so-called systematic redshift of transition wavelength, e.g., in case of the HOMO-LUMO transition [Dev2012]. This is demonstrated in Section 2.3. All these data are in accordance with conclusions in previous studies [41,42].

## 4. Conclusions

The structure of bikaverin molecules in chloroform solution was studied using DFT at the B3LYP/6-311G(2d,p) level. The lowest-energy state (state 1) was found and compared with data from a study of bikaverin single-crystal chloroform solvate. The main difference in geometry was the orientation of the MeO group attached to the benzene ring (ring 4). Moreover, the predicted bond and end lengths were in fairly good qualitative and quantitative agreement. A study of the rotational conformers revealed the presence of a low energy state, coinciding with the experimental geometry of molecule in the bikaverin chloroform solvate (state 2). Presumably, stabilization of this geometry is due to the environment forming hydrogen bonds in the crystal. In addition to examining the Me-O group rotation, scans of the total potential energy during rotation of the OH groups along the corresponding C_ar_-O bond were also made. Within the framework of the used approach, a low energy state with opposite orientation of the hydroxyl groups in hydroquinone (state 5) is predicted. In this case, one of the hydroxyl groups was directed toward the carbonyl group of the γ-pyrone another hydroxyl group is the same oriented as in state 1. The possibility of tautomers was also investigated. Three tautomeric states were found; the lowest energy is the state where two hydrogen atoms of the hydroquinone moiety simultaneously transfer and form a bond with the oxygens of carbonyl groups of the quinone moiety (state 7). This is analogous to the previously predicted tautomerism in naphthazarin. Within the framework of the presented work, it was shown that states 2, 5 and 7 are higher in energy than state 1 by only 0.74, 0.32 and 1 kcal/mol, respectively. The symmetry of the molecules in all four states is C_s_.

A theoretical study of the vibrational properties of states 1, 2, 5 and 7 was carried out. For the most stable state 1, the most IR active peaks were interpreted. The obtained theoretical result was compared with the experimental peaks given in other earlier studies. Current study shows that IR absorption spectroscopy is a sensitive method for differentiating states by different types of vibrations localized near the C-OH---O=C fragments of the molecule. The most suitable peaks for differentiating states 1, 2 and 5 are in the ranges of 1494–1700 cm^−1^ with δ(COH) and ν(C=O) atomic displacements, 1100–1494 cm^−1^ with δ(COH) atomic displacements, as well as vibrational modes in 375–900 cm^−1^ with δ(C-C=O) and tors (CCOH) atomic displacements. This sensitivity to polar groups may also be useful in the study of bikaverin derivatives in biological processes.

Modeling of the electron absorption properties was performed using the TD-DFT approach for vertical singlet-singlet transitions in the practically important 200–600 nm region. The observed transitions for different states were interpreted based on the molecular orbitals involved. It was shown that the longest-wavelength transition occurs between HOMO-LUMO frontier orbitals and is sensitive to the geometry of the molecule.

The obtained theoretical results on the structure of bikaverin may be further useful in the study of binding bikaverin in molecular docking and in the tasks of designing new drugs for pharmaceutical purposes.

## Figures and Tables

**Table 1:** Comparison of selected bond lengths of bikaverin molecule in states 1, 2, 5 and 7 with experimental data from [1].

		Theory				Experiment
		State 1	State 2	State 5	State 7	
	**Bond label**	**Bond length, Å**				
Ring 1	C7C8	1.459	1.459	1.467	1.397	1.44(2)
	C8C9	1.492	1.492	1.485	1.433	1.51(2)
	C9C10	1.353	1.353	1.347	1.376	1.33(2)
	C10C11	1.454	1.454	1.469	1.412	1.44(2)
	C11C6	1.457	1.456	1.481	1.401	1.47(2)
	C8O36	1.236	1.236	1.236	1.331	1.23(2)
	C9O26	1.331	1.331	1.337	1.341	1.32(2)
	O26C27	1.433	1.433	1.431	1.431	1.49(2)
	C11O35	1.250	1.250	1.226	1.334	1.25(2)
Ring 2	C1C4	1.425	1.426	1.414	1.490	1.41(2)
	C4C7	1.391	1.391	1.398	1.445	1.40(2)
	C7C6	1.421	1.421	1.428	1.423	1.44(2)
	C6C5	1.402	1.403	1.399	1.456	1.38(2)
	C5C2	1.435	1.435	1.436	1.485	1.45(2)
	C4O31	1.333	1.333	1.336	1.239	1.34(2)
	C5O33	1.325	1.325	1.329	1.243	1.35(2)
Ring 3	C15O38	1.368	1.369	1.364	1.371	1.38(2)
	O38C1	1.345	1.344	1.348	1.334	1.37(2)
	C1C2	1.386	1.386	1.383	1.361	1.36(2)
	C2C3	1.491	1.492	1.470	1.492	1.47(2)
	C3C13	1.473	1.474	1.455	1.475	1.47(2)
	C3O37	1.222	1.223	1.245	1.221	1.22(2)
Ring 4	C15C13	1.399	1.404	1.405	1.398	1.39(2)
	C13C14	1.427	1.420	1.429	1.427	1.39(2)
	C14C16	1.380	1.388	1.377	1.380	1.45(2)
	C16C18	1.406	1.402	1.408	1.406	1.43(2)
	C18C17	1.386	1.389	1.386	1.386	1.37(2)
	C17C15	1.390	1.382	1.389	1.390	1.41(2)
	C14C39	1.507	1.507	1.506	1.507	1.54(2)
	C18O21	1.349	1.350	1.346	1.348	1.41(2)
	O21C22	1.429	1.429	1.431	1.430	1.49(2)
	**Angle label**	**Angle, degree**				
Ring 1	C7C8C9	117.50	117.49	118.16	119.16	118.3(15)
	C8C9C10	120.78	120.76	120.33	119.64	120.6(15)
	C9C10C11	122.16	122.18	123.40	121.09	121.9(15)
	C10C11C6	119.00	118.99	117.8	120.47	120.5(16)
	C11C6C7	119.48	119.51	118.98	118.42	117.8(15)
	C6C11O35	121.69	121.71	123.63	122.27	117.8(15)
	C10C11O35	119.31	119.29	118.49	117.26	121.7(15)
	C7C8O36	122.52	122.54	122.34	123.10	122.4(14)
	C9C8O36	119.98	119.97	119.5	117.74	119.2(15)
	C8C9O26	112.37	112.40	112.49	114.66	111.5(14)
	C9O26C27	118.13	118.15	117.73	118.31	115.7(12)
Ring 2	C1C4C7	118.32	118.36	118.10	116.73	118.1(15)
	C4C7C6	120.36	120.34	121.60	120.13	119.6(15)
	C7C6C5	120.50	120.49	119.09	121.77	120.2(14)
	C6C5C2	119.89	119.93	119.68	118.38	120.3(15)
	C1C4O31	117.84	117.79	117.57	119.6	118.3(13)
	C4C7C8	118.56	118.59	117.16	118.65	119.8(14)
	C7C4O31	123.85	123.84	124.33	123.64	123.5(15)
	C2C5O33	119.62	119.61	118.72	121.23	120.0(14)
	C6C5O33	120.49	120.45	121.60	120.39	119.7(13)
	C5C6C11	120.01	120.00	121.93	119.81	122.0(15)
Ring 3	C15O38C1	120.65	120.67	120.44	120.37	118.8(12)
	O38C1C4	113.71	113.66	115.41	111.43	112.7(13)
	O38C1C2	123.34	123.46	122.72	124.22	123.1(14)
	C3C2C1	119.01	119.06	119.27	119.24	120.8(14)
	C2C3C13	115.36	115.34	116.62	114.86	114.7(14)
	C2C3O37	121.87	121.89	120.24	122.37	123.7(15)
Ring 4	C13C14C16	118.93	119.37	118.79	118.85	121.2(15)
	C39C14C13	122.82	122.76	122.66	122.78	124.2(14)
	C14C16C18	122.19	121.64	122.17	122.32	115.4(15)
	C16C18O21	115.59	124.37	115.45	115.60	125.4(15)
	C18C17C15	117.83	118.45	117.96	117.66	115.1(15)
	C17C15C13	123.98	123.58	123.52	124.24	124.7(15)
	C17C15O38	113.93	114.47	114.11	113.99	112.6(14)
	**Hydrogen bonds and contacts label**	**Hydrogen bonds and contacts length, Å**				
	O33H34	1.003	1.003	0.999	1.613	--
	H34O35	1.594	1.593	--	1.001	--
	O37H40	2.532	2.523	2.544	2.537	--
	O37H41	2.532	2.523	2.544	2.537	--
	O31H32	0.993	0.993	0.994	1.667	--
	H32O36	1.665	1.668	1.633	0.993	--
	H34O37	--	--	1.600	--	--

**Table 2:** Selected UV–vis singlet-singlet vertical transitions for bikaverin molecule in chloroform solution for states 1, 2, 5 and 7.

Theory				
State	Excited State №	Orbitals with > 14% Contribution (Percent)	Oscillator Strength	Wavelength, nm (Energy, eV)
1	1	99HOMO -> 100LUMO (98)	0.2620	528.48 (2.3461)
	6	95HOMO-4 -> 100 LUMO (88)	0.0375	375.26 (3.3040)
	10	93HOMO-6 -> 100 LUMO (88)	0.1532	306.98 (4.0388)
	11	98 HOMO-1 -> 101 LUMO+1 (58) 96 HOMO-3 -> 101 LUMO+1 (28)	0.3014	284.92 (4.3515)
	13	99 HOMO -> 102 LUMO+2 (82)	0.3796	272.63 (4.5477)
	17	95 HOMO-4 -> 101 LUMO+1 (58) 91 HOMO-8 -> 100 LUMO (17) 99 HOMO -> 103 LUMO+2 (14)	0.3104	247.34 (5.0127)
	24	93 HOMO-6 -> 101 LUMO+1 (78)	0.1960	228.71 (5.4210)
	28	98 HOMO-1 -> 103 LUMO+3 (61)	0.2118	219.95 (5.6368)
	37	96 HOMO-3 -> 104 LUMO+4 (52) 95 HOMO-4 -> 103 LUMO+3 (17)	0.2380	203.07 (6.1056)
	39	95HOMO-4 -> 103 LUMO+3 (63) 96 HOMO-3 -> 104 LUMO+4 (18)	0.2391	201.16 (6.1635)
2	1	99 HOMO -> 100 LUMO (98)	0.2532	528.05 (2.3480)
	6	95 HOMO-4 -> 100 LUMO (88)	0.0436	375.32 (3.3034)
	10	93 HOMO-6 -> 100 LUMO (89)	0.1493	305.10 (4.0637)
	11	98 HOMO-1 -> 101 LUMO+1 (82)	0.3990	292.09 (4.2447)
	13	99 HOMO -> 102 LUMO+2 (83)	0.2659	272.37 (4.5520)
	17	95 HOMO-4 -> 101 LUMO+1 (58) 91 HOMO-8 -> 100 LUMO (22)	0.2873	248.15 (4.9962)
	24	93 HOMO-6 -> 101 LUMO+1 (69) 98 HOMO-1 -> 103 LUMO+3 (15)	0.1865	229.34 (5.4062)
	37	96 HOMO-3 -> 104 LUMO+4 (62) 93 HOMO6 -> 102 LUMO+2 (16)	0.1838	203.79 (6.0840)
	39	95 HOMO-4 -> 103 LUMO+2 (80)	0.2313	200.84 (6.1732)
5	1	99 HOMO -> 100 LUMO (99)	0.2367	521.28 (2.3784)
	3	97 HOMO-2 -> 100 LUMO (59) 96 HOMO-3 -> 100 LUMO (30)	0.0572	400.84 (3.0931)
	7	99 HOMO -> 101 LUMO+1 57	0.0453	361.40 (3.4307)
	11	97 HOMO-2 -> 101 LUMO+1 (57) 93 HOMO-6 -> 100 LUMO (14)	0.3715	289.30 (4.2856)
	14	99 HOMO -> 102 LUMO+2 (36) 97 HOMO-2 -> 101 LUMO+1 (20)	0.4427	276.00 (4.4922)
	22	97 HOMO-2 -> 102 LUMO+2 (58) 93 HOMO-6 -> 101 LUMO+1 (22)	0.1756	232.11 (5.3416)
	24	90 HOMO-9 -> 100 LUMO (68) 96 HOMO-3 -> 102 LUMO+2 (25)	0.1320	228.58 (5.4240)
	29	97 HOMO-2 -> 103 LUMO+3 (51) 96 HOMO-3 -> 103 LUMO+3 (14)	0.1956	214.03 (5.7928)
	35	95 HOMO-4 -> 103 LUMO+3 (40) 91 HOMO-8 -> 101 LUMO+1 (26)	0.2172	204.93 (6.0501)
7	1	99 HOMO -> 100 LUMO (97)	0.1989	543.42 (2.2815)
	3	97 HOMO-2 -> 100 LUMO (94)	0.0357	446.38 (2.7776)
	5	95 HOMO-4 -> 100 LUMO (92)	0.0771	379.60 (3.2662)
	9	99 HOMO -> 101 LUMO+1 (89)	0.0764	325.86 (3.8049)
	10	93 HOMO-6 -> 100 LUMO (80)	0.0948	305.59 (4.0572)
	12	96 HOMO-3 -> 101 LUMO+1 (79)	0.2689	275.96 (4.4929)
	18	95 HOMO-4 -> 101 LUMO+1 (78)	0.2328	248.34 (4.9925)
	26	97 HOMO-4 -> 103 LUMO+3 (37) 93 HOMO-6 -> 101 LUMO+1 (26)	0.2214	222.98 (5.5603)
	37	95 HOMO-4 -> 103 LUMO+3 (72)	0.3487	205.53 (6.0324)

**Table 3:** Energies of frontier orbitals as well as derived parameters for states 1, 2, 5 and 7.

State	1	2	5	7
Energy of HOMO, Ha (eV)	−0.22583 (−6.145)	−0.2258 (−6.144)	−0.22271 (−6.060)	−0.22457 (−6.111)
Energy of LUMO, Ha (eV)	−0.12547 (−3.414)	−0.12508 (−3.404)	−0.12000 (−3.265)	−0.12632 (−3.437)
HOMO-LUMO gap, Ha (eV)	0.10036 (2.731)	0.1005 (2.735)	0.10271 (2.795)	0.09825 (2.674)
Ionization potential, eV	6.145	6.144	6.060	6.111
Electron affinity, eV	3.414	3.404	3.265	3.437
Electronegativity, eV	4.780	4.774	4.663	4.774
Chemical hardness, eV	1.366	1.370	1.398	1.337
Electrophilicity, eV	8.365	8.318	7.778	8.523
Chemical softness, eV	0.366	0.365	0.358	0.374

## Data Availability

The original contributions presented in this study are included in the article/Supplementary Materials. Further inquiries can be directed to the corresponding author.

## Supplementary Materials

The following supporting information can be downloaded at: https://www.mdpi.com/article/10.3390/molecules30234634/s1, Tables S1.1: Optimized geometry of state 1; Tables S1.2: Optimized geometry of state 2; Tables S1.3: Optimized geometry of state 5; Tables S1.4: Optimized geometry of state 7; Table S2.1: Calculated IR absorbance and Raman spectra for state 1; Table S2.2: Calculated IR absorbance and Raman spectra for state 2; Table S2.3: Calculated IR absorbance and Raman spectra for state 5; Table S2.4: Calculated IR absorbance and Raman spectra for state 7.
