# Supplementary material for: Theoretical Investigation of Structural and Optical Peculiarities of Bikaverin Fungal Pigment in Chloroform Solution
Source: Molecules. 2025 Dec 2;30(23):4634. doi: 10.3390/molecules30234634 (PMC12693441; doi:10.3390/molecules30234634)
Supplement: Supplementary file 1 [file molecules-30-04634-s001.zip › molecules-3937466-supplementary.pdf]

Supporting information

for

Theoretical investigation of structural and optical peculiarities of bikaverin fungal pigment in chloroform solution.

Anastasia Povolotckaia [1,2], Dmitrii Pankin [2], Sergey Belousov [3], Andey Boyko [4], Sergey Akulov [1], Evgenii Borisov [2], Anatoly Gulyaev [1], Sergey Gudkov [1,5,6], Andrey Izmailov [1], Maksim Moskovskiy [1]

[1] Federal Scientific Agroengineering Center VIM, 109428 Moscow, Russia; tomasss1086@mail.ru (A.G.), maxmoskovsky74@yandex.ru (M.M.), serg.akulov.76@mail.ru (S.A.), s_makariy@rambler.ru (S.G.), anatoly10121986@gmail.com (A.I.)
[2] Center for Optical and Laser Materials Research, St. Petersburg State University, 198504 St. Petersburg, Russia; dmitrii.pankin@spbu.ru (D.P.), eugene.borisov@spbu.ru (E.B.)
[3] Department of Processes and Machines in Agribusiness, Kuban State Agrarian University Named after I.T. Trubilin, 350044 Krasnodar, Russia; sergey_belousov_87@mail.ru (S.B.)
[4] Don State Technical University, 346780 Rostov-on-Don, Russia; andreyboi@yandex.ru (A.B.)
[5] 1- Prokhorov General Physics Institute of the Russian Academy of Sciences, 119991 Moscow, Russia, s_makariy@rambler.ru
[6] Bauman Moscow State Technical University, 5 2nd Baumanskaya St., 105005 Moscow, Russia 3-Federal Scientific Agro-Engineering Center VIM, 1st Institutskiy proezd 5, 109428 Moscow, Russia
* Correspondence: anastasia.povolotckaia@spbu.ru

1. Optimized geometry of states.

Tables S1.1. Optimized geometry of state 1.

C	0.00000000	0.50939200	0.00000000
C	-0.67044600	-0.70361100	0.00000000
C	-2.16173500	-0.70581800	0.00000000
C	1.42146700	0.60914500	0.00000000
C	0.11298800	-1.90642000	0.00000000
C	1.51329300	-1.82836200	0.00000000
C	2.16521200	-0.56597500	0.00000000
C	3.62112300	-0.47003500	0.00000000
C	4.39563700	-1.74555200	0.00000000
C	3.76142700	-2.94070400	0.00000000
C	2.31117100	-3.04742100	0.00000000
H	4.29715300	-3.87938400	0.00000000
C	-2.79475100	0.62459900	0.00000000
C	-4.20867000	0.81851300	0.00000000
C	-1.99194900	1.76985000	0.00000000
C	-4.70583900	2.10546700	0.00000000
C	-2.49000700	3.06766400	0.00000000
C	-3.86611100	3.23265800	0.00000000
H	-5.77495200	2.27636000	0.00000000
H	-1.79272200	3.89126200	0.00000000
O	-4.49377200	4.42680400	0.00000000
C	-3.70047300	5.61561300	0.00000000
H	-4.40768500	6.44068700	0.00000000
H	-3.07487000	5.66881200	0.89396700
H	-3.07487000	5.66881200	-0.89396700
O	5.71084900	-1.53965000	0.00000000
C	6.57381200	-2.68375200	0.00000000
H	7.58451700	-2.28686100	0.00000000
H	6.40892400	-3.28806000	0.89413300
H	6.40892400	-3.28806000	-0.89413300
O	1.95987100	1.82856100	0.00000000
H	2.94281300	1.68758200	0.00000000
O	-0.49495700	-3.08413900	0.00000000
H	0.24139500	-3.76476300	0.00000000

O	1.78080600	-4.17955700	0.00000000
O	4.21566000	0.61349900	0.00000000
O	-2.80566100	-1.74489900	0.00000000
O	-0.62566600	1.69990900	0.00000000
C	-5.18975100	-0.32509400	0.00000000
H	-5.05244100	-0.96688900	-0.87055900
H	-5.05244100	-0.96688900	0.87055900
H	-6.20917300	0.06223600	0.00000000

Tables S1.2. Optimized geometry of state 2.

C	0.00000000	0.55820200	0.00000000
C	0.45995000	-0.74955000	0.00000000
C	1.93038500	-1.00032600	0.00000000
C	-1.38626800	0.89095900	0.00000000
C	-0.51190500	-1.80565900	0.00000000
C	-1.88023400	-1.49755400	0.00000000
C	-2.31433900	-0.14487500	0.00000000
C	-3.73468500	0.19034800	0.00000000
C	-4.70931300	-0.94003400	0.00000000
C	-4.28070400	-2.22330600	0.00000000
C	-2.86803300	-2.56790300	0.00000000
H	-4.96358300	-3.06097900	0.00000000
C	2.77614900	0.20678800	0.00000000
C	4.19549900	0.16330200	0.00000000
C	2.17248500	1.47469300	0.00000000
C	4.91303500	1.35154600	0.00000000
C	2.88334000	2.65949800	0.00000000
C	4.27136000	2.59834500	0.00000000
H	5.99199900	1.29921900	0.00000000
H	2.36740300	3.60938300	0.00000000
O	4.91358100	3.78609300	0.00000000
C	6.34286200	3.80118000	0.00000000
H	6.62286300	4.85129200	0.00000000
H	6.74161800	3.31679900	0.89400700
H	6.74161800	3.31679900	-0.89400700
O	-5.97278700	-0.52058400	0.00000000
C	-7.01254100	-1.50693700	0.00000000
H	-7.94409200	-0.94904100	0.00000000
H	-6.94960300	-2.13020400	-0.89413300
H	-6.94960300	-2.13020400	0.89413300
O	-1.71559200	2.18234200	0.00000000
H	-2.70818600	2.20619700	0.00000000
O	-0.10757700	-3.06752800	0.00000000
H	-0.94625800	-3.61765300	0.00000000
O	-2.53218000	-3.77240800	0.00000000
O	-4.14202800	1.35708200	0.00000000
O	2.39259300	-2.13208000	0.00000000
O	0.81215000	1.62954700	0.00000000
C	4.97175000	-1.12811200	0.00000000
H	4.72787300	-1.73806700	0.87009500
H	4.72787300	-1.73806700	-0.87009500
H	6.04205600	-0.91880000	0.00000000

Tables S1.3. Optimized geometry of state 5.

C	0.24792200	-0.48175600	-0.00013200
C	0.22832800	0.90123900	-0.00007800
C	1.50014500	1.63787400	-0.00005100
C	-0.94244500	-1.24542700	-0.00011000
C	-1.02911100	1.59375000	-0.00001300
C	-2.22208400	0.86335500	0.00004400
C	-2.16269200	-0.56298500	-0.00002600
C	-3.38436500	-1.37552900	0.00005500
C	-4.69302000	-0.67359100	0.00006100
C	-4.74283400	0.67271800	0.00015400
C	-3.54685200	1.52654100	0.00010500
H	-5.67750100	1.21564200	0.00022400

C	2.71612800	0.83921700	-0.00006200
C	4.03365200	1.39353600	0.00000100
C	2.60066900	-0.56098600	-0.00013800
C	5.11288500	0.53845700	-0.00001700
C	3.69182600	-1.42067700	-0.00012300
C	4.96030800	-0.86111700	-0.00007300
H	6.12129000	0.93214200	0.00004600
H	3.51250800	-2.48472800	-0.00024000
O	6.10552100	-1.56764000	-0.00006600
C	6.03120700	-2.99654600	0.00040000
H	7.06084600	-3.34324400	0.00099200
H	5.52022500	-3.35991900	0.89449600
H	5.52103500	-3.36051700	-0.89392200
O	-5.72729700	-1.52025300	-0.00000200
C	-7.04520000	-0.96185400	-0.00005800
H	-7.72332200	-1.81026900	-0.00035400
H	-7.20674700	-0.35524300	0.89363100
H	-7.20654200	-0.35481300	-0.89348800
O	-0.82340300	-2.57607700	-0.00013900
H	-1.75378000	-2.92531000	-0.00002000
O	-1.02620800	2.92306500	-0.00000600
H	-0.06541500	3.19663800	-0.00002700
O	-3.69701200	2.74352000	0.00010700
O	-3.35649600	-2.61134400	0.00011300
O	1.50372100	2.88324800	-0.00000500
O	1.39214500	-1.19417500	-0.00021300
C	4.29112800	2.87758300	0.00008100
H	3.84601900	3.35827900	-0.87161400
H	3.84558700	3.35828800	0.87153200
H	5.36444800	3.06748900	0.00032100

Tables S1.4. Optimized geometry of state 7.

C	0.28428300	-0.40889400	-0.02319600
C	0.25936600	0.95219200	-0.01993400
C	1.54693000	1.70469200	-0.06067000
C	-0.93047900	-1.27189500	-0.02590000
C	-1.05669500	1.63984100	0.01820300
C	-2.26328900	0.82544700	0.01489600
C	-2.20749300	-0.59639800	-0.01063000
C	-3.37265300	-1.36663500	-0.01550000
C	-4.64529400	-0.70781400	0.00360400
C	-4.69961900	0.66682500	0.03058900
C	-3.52021900	1.44297500	0.03914700
H	-5.64194100	1.19469500	0.04704800
C	2.75815000	0.86365400	-0.02283100
C	4.08242000	1.39367200	-0.00282400
C	2.62853300	-0.52826700	-0.01459400
C	5.14923600	0.51899500	0.02301600
C	3.69960600	-1.41320800	0.00730600
C	4.97735700	-0.87645900	0.02671300
H	6.16325000	0.89827900	0.04081300
H	3.49846100	-2.47346400	0.00972000
O	6.11294500	-1.60328000	0.05072400
C	6.01082700	-3.02915100	0.05586600
H	7.03311300	-3.39692100	0.07570900
H	5.47739100	-3.37975700	0.94227800
H	5.50894100	-3.38845600	-0.84535900
O	-5.70265800	-1.53275500	-0.00558200
C	-7.01235100	-0.95729100	0.01410700
H	-7.70024800	-1.79782900	0.00255400
H	-7.16349400	-0.36751200	0.92081900
H	-7.17542000	-0.33325400	-0.86723800
O	-0.80568700	-2.50401700	-0.04322800
H	-2.40756700	-2.96444900	-0.04524100
O	-1.13495400	2.87948700	0.05836600
H	-2.72851500	3.12985500	0.07544100

O	-3.66168700	2.76879300	0.06978600
O	-3.36420100	-2.69742000	-0.03630100
O	1.59017200	2.92350700	-0.12816100
O	1.40104900	-1.13892500	-0.03231500
C	4.36906600	2.87274900	-0.00485900
H	3.97234000	3.35250500	-0.89983900
H	3.89243000	3.37159700	0.83920100
H	5.44533200	3.04139500	0.04274000

2. Calculated vibrational properties for various states.

Table S2.1. Calculated IR absorbance and Raman spectra for state 1.

Vibrational mode number	Calculated frequency of vibrational mode, cm^{-1}	Scaled calculated frequency of vibrational mode, cm^{-1}	Infrared activity, KM/Mole	Raman activity, A^4/AMU
1	21.64	21.21	2.5612	0.4661
2	34.86	34.17	0.0053	0.0291
3	49.69	48.70	0.0762	0.2322
4	74.97	73.47	1.1454	0.6463
5	97.14	95.20	1.0937	1.9822
6	98.46	96.49	8.7115	0.8986
7	110.35	108.14	1.3886	1.6734
8	128.96	126.38	0.0083	0.3051
9	144.97	142.07	0.1861	1.0578
10	169.05	165.67	0.8287	1.8608
11	170.03	166.63	6.0107	1.0525
12	194.20	190.32	0.1370	0.0357
13	203.19	199.13	3.4241	5.9595
14	204.86	200.76	0.0661	1.1854
15	208.64	204.47	0.9144	0.1865
16	223.83	219.35	0.2406	1.8730
17	233.49	228.82	1.5789	2.8275
18	254.89	249.79	1.1354	0.5379
19	261.59	256.36	0.2144	2.6808
20	291.03	285.21	0.0188	1.3639
21	296.82	290.88	9.5931	1.5815
22	311.98	305.74	20.1748	3.8000
23	322.82	316.36	4.5753	4.7619
24	335.74	329.02	0.6897	4.9085
25	366.57	359.23	16.0791	11.8315
26	375.24	367.73	1.2425	25.6298
27	408.40	400.23	13.3024	61.0822
28	422.48	414.03	0.3553	0.7711
29	442.91	434.06	3.9358	26.0262
30	446.44	437.51	21.1637	65.0970
31	465.90	456.58	1.1314	0.4774
32	471.83	462.39	14.4854	26.0681
33	482.01	472.37	5.7074	17.2604
34	502.04	492.00	101.8245	8.2030
35	529.72	519.12	2.6695	14.1156
36	551.13	540.11	0.8510	0.4854
37	561.41	550.19	16.4868	21.4006
38	576.32	564.79	4.2762	132.6301
39	582.90	571.25	0.0055	4.9971
40	604.05	591.97	1.7772	10.5550

41	620.89	608.47	9.0578	20.4307
42	643.43	630.56	0.1448	0.8193
43	663.42	650.15	3.4640	1.6356
44	712.39	698.15	39.8482	42.6698
45	713.76	699.48	6.8239	1.1267
46	731.95	717.32	6.9959	5.5850
47	739.28	724.50	4.1406	0.5775
48	763.68	748.41	1.2877	2.2499
49	764.41	749.12	0.0368	1.6390
50	804.79	788.69	9.8450	2.1141
51	811.36	795.13	27.0181	11.8426
52	826.19	809.67	8.9456	0.0415
53	835.40	818.69	87.7155	0.6584
54	851.10	834.08	35.4009	0.0475
55	881.74	864.11	25.7671	0.3772
56	886.28	868.56	33.0685	3.6386
57	917.03	898.69	21.7862	42.6152
58	922.53	904.08	108.4440	1.8417
59	950.47	931.47	36.6507	53.5858
60	973.34	953.87	48.1942	109.5968
61	997.97	978.01	67.3700	22.0936
62	1007.10	986.95	11.3714	49.4509
63	1043.84	1022.96	188.0632	187.9320
64	1058.91	1037.73	105.3405	8.9608
65	1062.87	1041.61	4.5457	0.0655
66	1083.34	1061.67	1.2581	58.6056
67	1148.93	1125.95	193.8666	387.3832
68	1164.10	1140.82	702.9915	89.7216
69	1169.42	1146.03	1.6312	2.3344
70	1170.22	1146.82	1.7073	1.7551
71	1185.79	1162.08	26.8803	23.9967
72	1208.06	1183.90	63.7799	46.6807
73	1224.68	1200.18	106.2110	41.1300
74	1231.30	1206.68	106.9821	52.0321
75	1245.87	1220.95	878.1206	719.6576
76	1270.36	1244.95	221.9606	74.9718
77	1287.17	1261.43	128.2141	1549.7256
78	1306.64	1280.51	55.0982	80.4569
79	1312.04	1285.80	222.6792	118.8096
80	1339.08	1312.30	1061.8349	880.4144
81	1376.25	1348.72	295.7038	278.7341
82	1394.33	1366.45	305.7797	199.7426
83	1406.36	1378.24	93.7302	217.8233
84	1410.22	1382.02	112.2856	2959.7142
85	1417.65	1389.30	2.1970	62.2273
86	1437.58	1408.83	879.5212	96.5522
87	1446.78	1417.84	43.0051	45.6396
88	1464.69	1435.40	140.6477	835.7390
89	1467.40	1438.05	9.1997	19.8784
90	1474.45	1444.96	63.9651	479.2419
91	1478.02	1448.46	22.3218	71.9244
92	1484.85	1455.15	259.2478	122.3765

93	1488.68	1458.91	13.5811	22.3834
94	1490.12	1460.32	12.0373	23.9810
95	1495.76	1465.84	46.2513	8.8248
96	1501.40	1471.38	12.2819	12.7829
97	1505.87	1475.75	3.9436	83.3433
98	1513.86	1483.58	83.9926	64.7893
99	1586.09	1554.37	44.0346	744.0878
100	1607.94	1575.78	258.7427	103.9864
101	1622.05	1589.60	808.7694	144.3107
102	1633.98	1601.30	184.2264	386.4913
103	1646.80	1613.86	871.7400	305.0952
104	1652.33	1619.29	134.8778	125.1940
105	1683.39	1649.73	122.4830	812.1026
106	1702.71	1668.66	380.1322	940.5546
107	3022.13	2961.69	55.9257	233.4729
108	3031.41	2970.79	55.5186	308.6164
109	3044.62	2983.73	31.0054	361.5580
110	3047.58	2986.63	371.4838	902.0072
111	3087.12	3025.37	32.2630	88.3172
112	3099.81	3037.82	24.2969	99.4650
113	3103.80	3041.73	10.3956	116.4937
114	3113.06	3050.80	33.9148	177.5279
115	3146.81	3083.87	22.9763	211.3772
116	3159.47	3096.28	16.3063	193.9605
117	3185.81	3122.09	9.7255	298.5632
118	3211.78	3147.55	5.6603	364.4794
119	3227.06	3162.52	0.8819	133.3695
120	3244.87	3179.97	184.5698	538.3731

Table S2.2. Calculated IR absorbance and Raman spectra for state 2.

Vibrational mode number	Calculated frequency of vibrational mode, cm^{-1}	Scaled calculated frequency of vibrational mode, cm^{-1}	Infrared activity, KM/Mole	Raman activity, A^4/AMU
1	15.78	15.47	3.9018	0.3437
2	34.62	33.92	0.0564	0.0015
3	51.09	50.07	0.2738	0.1825
4	66.08	64.76	0.1898	0.1973
5	93.44	91.57	7.4943	2.4127
6	97.71	95.76	2.2111	1.1469
7	111.42	109.19	2.2931	0.5623
8	127.28	124.73	0.0527	0.0339
9	148.51	145.54	0.0609	1.3540
10	166.50	163.17	5.8587	0.9363
11	174.77	171.28	4.6940	0.3568
12	184.37	180.68	0.0983	1.6633
13	188.99	185.21	0.0044	0.2031
14	211.07	206.84	1.1763	0.1922
15	216.62	212.28	1.0896	3.2534
16	219.89	215.49	0.1206	5.7087
17	234.94	230.25	0.0115	1.0124
18	245.76	240.85	1.6872	0.1809
19	253.57	248.50	0.6527	3.4185

20	270.25	264.85	1.5609	5.9424
21	293.10	287.23	0.0018	0.6861
22	314.28	307.99	21.4402	3.5485
23	333.87	327.19	0.6298	4.9072
24	337.06	330.32	11.6987	0.4020
25	376.19	368.67	6.2139	7.5300
26	390.78	382.96	1.5307	63.9672
27	410.20	401.99	6.0018	14.8797
28	421.26	412.84	20.5640	15.2580
29	421.81	413.37	0.3092	0.7429
30	449.11	440.13	21.2811	82.4693
31	464.47	455.18	1.0480	0.4754
32	470.81	461.39	19.2026	23.6469
33	480.62	471.01	3.0544	23.5476
34	506.28	496.15	61.3393	8.9674
35	533.21	522.54	6.7422	8.4223
36	552.20	541.16	1.1594	1.4080
37	565.23	553.92	44.7749	71.0285
38	577.87	566.31	29.5560	87.3299
39	582.39	570.74	0.0001	3.1991
40	605.81	593.69	21.6559	20.0451
41	622.82	610.36	2.6086	11.1020
42	643.27	630.40	0.0266	1.6143
43	663.18	649.92	3.4743	2.2593
44	692.38	678.53	10.8316	32.0977
45	713.49	699.22	6.2698	1.1933
46	733.12	718.45	3.3534	6.3744
47	739.19	724.41	4.2797	0.8273
48	763.44	748.17	7.5461	7.6874
49	764.03	748.75	0.0023	1.1758
50	804.14	788.06	7.6812	2.1851
51	811.93	795.69	19.6754	12.6235
52	827.69	811.14	19.4644	0.0173
53	836.21	819.49	82.5197	0.7016
54	857.19	840.04	0.6562	0.3264
55	868.07	850.70	57.6152	0.0984
56	881.45	863.82	35.5025	3.7644
57	916.52	898.19	35.4561	22.9295
58	922.33	903.88	107.7311	1.4990
59	958.59	939.41	68.6059	47.0575
60	967.22	947.87	5.9002	166.5121
61	996.83	976.89	74.8210	24.1415
62	1012.93	992.67	43.7488	35.7792
63	1040.80	1019.98	79.2052	149.8681
64	1050.48	1029.48	92.7977	27.1640
65	1061.58	1040.35	4.8248	0.0640
66	1084.96	1063.26	28.0335	90.2394
67	1143.37	1120.51	252.4960	370.4537
68	1163.83	1140.55	803.3749	77.9686
69	1168.00	1144.64	1.7536	1.7025
70	1169.64	1146.25	1.4172	2.0685
71	1192.82	1168.96	116.3564	64.6950

72	1204.42	1180.33	17.5665	48.6007
73	1212.90	1188.64	137.2385	5.3422
74	1230.79	1206.18	2.6470	56.2724
75	1244.15	1219.26	1096.6348	758.4162
76	1278.83	1253.25	87.0614	439.1754
77	1287.29	1261.55	109.5943	1152.6368
78	1309.56	1283.37	46.3582	106.5821
79	1327.51	1300.96	534.5050	254.1282
80	1335.80	1309.08	676.8774	891.4999
81	1357.64	1330.48	347.1487	160.6164
82	1392.75	1364.90	315.9863	181.7920
83	1406.27	1378.15	20.1940	1356.6193
84	1408.16	1380.00	317.5195	1136.2268
85	1416.94	1388.60	6.7730	93.7485
86	1436.65	1407.92	813.0762	81.8159
87	1440.96	1412.14	115.5369	38.3352
88	1466.98	1437.64	290.9257	1280.4122
89	1467.93	1438.57	9.5707	19.1186
90	1476.46	1446.93	14.1357	11.7273
91	1477.99	1448.43	11.4484	235.1502
92	1487.78	1458.03	85.6068	29.4917
93	1487.87	1458.11	13.6904	22.4371
94	1490.77	1460.96	11.1264	26.1127
95	1493.09	1463.23	43.1984	5.4336
96	1501.18	1471.15	85.9292	14.8000
97	1506.65	1476.52	28.0148	206.3531
98	1519.76	1489.37	47.1693	10.8968
99	1584.96	1553.26	28.2583	744.8734
100	1607.44	1575.29	147.8953	111.6512
101	1621.75	1589.32	903.1161	175.3174
102	1632.26	1599.61	179.3359	280.4678
103	1644.17	1611.29	1058.4265	478.7217
104	1651.56	1618.53	43.3447	89.8403
105	1683.56	1649.89	128.2460	726.3238
106	1703.07	1669.01	366.7648	960.8454
107	3024.21	2963.72	73.6670	261.8638
108	3030.08	2969.48	56.5402	297.2104
109	3043.04	2982.18	380.1758	936.4980
110	3044.20	2983.32	23.3230	314.6318
111	3089.74	3027.94	32.8411	116.9887
112	3098.41	3036.45	24.6026	97.4647
113	3104.07	3041.99	9.4287	114.6692
114	3112.48	3050.23	34.4377	170.3581
115	3145.25	3082.34	21.8959	209.9849
116	3159.34	3096.15	16.1997	197.2793
117	3207.07	3142.93	9.6639	219.3555
118	3209.60	3145.41	5.8496	351.7474
119	3211.14	3146.92	0.0660	175.1403
120	3247.30	3182.36	184.6522	529.1777

Table S2.3. Calculated IR absorbance and Raman spectra for state 5.

Vibrational mode number	Calculated frequency of vibrational mode, cm^{-1}	Scaled calculated frequency of	Infrared activity, KM/Mole	Raman activity, A^4/AMU

		vibrational mode, cm^{-1}		
1	24.86	24.37	2.3769	0.3513
2	37.71	36.96	0.1226	0.3510
3	55.75	54.64	1.4287	0.0605
4	79.21	77.62	0.0160	0.0807
5	99.32	97.33	1.5849	3.0702
6	105.69	103.58	4.3319	0.2985
7	108.56	106.39	4.9239	1.0055
8	121.45	119.02	1.0464	0.1937
9	141.40	138.57	0.0014	1.1677
10	158.39	155.23	6.1731	1.6239
11	168.54	165.17	1.5966	4.5447
12	187.74	183.98	0.6144	0.1936
13	202.87	198.82	0.1278	0.7604
14	208.80	204.62	1.6714	1.4836
15	212.81	208.55	1.0030	0.3012
16	226.54	222.01	0.0884	1.8308
17	235.52	230.81	2.1087	3.5454
18	247.46	242.51	0.9810	0.2245
19	263.64	258.36	0.2068	3.7537
20	281.49	275.86	2.7487	7.0537
21	291.49	285.66	0.0006	0.4076
22	314.05	307.77	17.6380	6.4513
23	319.12	312.74	6.9501	3.5565
24	332.42	325.77	2.5102	5.3484
25	368.95	361.58	10.3208	38.9563
26	396.17	388.24	60.3989	32.8810
27	411.80	403.56	8.6473	30.6784
28	424.16	415.67	0.2620	1.2044
29	449.34	440.36	15.6131	70.3552
30	457.04	447.90	45.2738	41.0043
31	460.03	450.83	1.5281	0.2529
32	469.71	460.31	17.3756	19.2327
33	480.07	470.47	4.8254	61.2114
34	508.81	498.63	78.4442	21.8510
35	530.19	519.58	3.3782	7.1523
36	553.05	541.99	0.3804	0.3240
37	558.02	546.86	12.3116	22.7122
38	566.00	554.68	4.8779	51.8048
39	584.22	572.54	0.0022	5.2539
40	601.43	589.40	1.8123	9.4128
41	614.80	602.50	5.9879	51.3370
42	644.67	631.78	0.1236	0.8299
43	661.89	648.65	2.8342	1.5160
44	711.20	696.98	1.9123	1.2163
45	715.65	701.34	59.3173	80.7806
46	736.40	721.67	2.2012	0.2917
47	737.55	722.80	1.8594	0.3407
48	764.03	748.75	13.8454	10.4079
49	771.29	755.86	2.2219	3.1971
50	807.79	791.63	11.2332	1.6405
51	814.22	797.93	10.1040	3.6490

52	818.47	802.10	6.8142	0.1838
53	838.42	821.66	88.2563	0.6246
54	853.91	836.83	27.7703	0.1183
55	883.23	865.56	30.8731	0.5838
56	897.13	879.18	138.6392	0.8740
57	898.81	880.83	5.1146	3.7339
58	916.48	898.15	39.1286	1.3601
59	953.32	934.25	68.9264	10.1163
60	972.86	953.40	88.9817	33.5986
61	978.47	958.91	25.9668	10.4455
62	1008.60	988.42	14.0065	69.7624
63	1052.74	1031.68	184.7401	124.0229
64	1062.03	1040.79	195.6777	60.0690
65	1063.50	1042.23	4.4480	0.1075
66	1086.28	1064.55	22.2296	62.3054
67	1145.61	1122.70	280.2021	227.8326
68	1166.13	1142.81	526.2062	71.7588
69	1170.35	1146.95	1.4753	2.1865
70	1171.47	1148.04	1.6764	1.7803
71	1187.59	1163.84	28.8485	2.8828
72	1208.73	1184.56	260.4489	26.9624
73	1224.75	1200.26	106.4461	19.6362
74	1234.25	1209.56	1174.8175	143.0037
75	1241.01	1216.19	9.2789	6.3146
76	1271.78	1246.34	323.3161	149.2894
77	1281.14	1255.52	111.5719	329.1990
78	1300.35	1274.34	64.5221	87.3957
79	1311.84	1285.60	199.8916	112.5148
80	1330.98	1304.36	751.9852	954.8013
81	1367.81	1340.46	84.9231	53.5955
82	1386.20	1358.47	145.9606	45.9862
83	1404.34	1376.25	20.8582	3402.0745
84	1417.43	1389.08	1.9276	36.7418
85	1419.99	1391.59	105.0841	343.1888
86	1431.15	1402.53	217.5706	1291.8610
87	1447.28	1418.33	166.0616	125.6503
88	1461.66	1432.43	196.5201	714.6370
89	1469.80	1440.40	10.1179	19.6194
90	1475.71	1446.20	77.3403	101.3635
91	1479.03	1449.45	16.0321	9.2959
92	1488.71	1458.94	523.4495	99.9768
93	1489.31	1459.52	12.9763	22.5107
94	1490.40	1460.59	12.3049	24.1614
95	1496.00	1466.08	21.9160	3.4790
96	1502.61	1472.56	16.7945	5.3936
97	1512.67	1482.42	157.8671	30.4240
98	1536.73	1506.00	611.5673	256.7102
99	1581.45	1549.82	62.7506	470.4221
100	1592.06	1560.22	579.1765	286.3946
101	1622.82	1590.36	490.1853	350.0539
102	1646.50	1613.57	671.1928	27.2880
103	1651.09	1618.07	392.0449	80.2354

104	1655.75	1622.64	29.8355	92.7536
105	1680.58	1646.97	198.2762	599.7063
106	1699.93	1665.93	315.2379	1551.6701
107	3026.59	2966.05	62.9708	158.1284
108	3026.74	2966.20	42.5663	373.8528
109	3046.25	2985.32	21.2236	330.8051
110	3093.09	3031.22	21.1548	107.5575
111	3093.14	3031.28	35.6608	78.0596
112	3103.17	3041.11	9.3093	114.3042
113	3114.62	3052.33	704.6673	245.1235
114	3119.51	3057.12	692.1480	1104.1075
115	3150.53	3087.52	20.1858	218.5582
116	3155.16	3092.06	17.7397	191.5237
117	3188.06	3124.30	6.6467	322.3194
118	3208.31	3144.14	6.6384	334.7351
119	3222.74	3158.28	252.7655	831.4221
120	3229.35	3164.76	1.3350	144.3036

Table S2.4. Calculated IR absorbance and Raman spectra for state 7.

Vibrational mode number	Calculated frequency of vibrational mode, cm^{-1}	Scaled calculated frequency of vibrational mode, cm^{-1}	Infrared activity, KM/Mole	Raman activity, A^4/AMU
1	19.77	19.37	2.6126	0.7447
2	36.40	35.67	0.1088	0.0269
3	53.16	52.10	0.0518	1.4747
4	76.34	74.81	1.3776	0.5424
5	93.37	91.51	2.0584	2.0972
6	95.80	93.89	2.0167	2.2791
7	107.40	105.25	7.0469	1.1696
8	119.81	117.42	0.0302	0.0365
9	158.37	155.20	0.1330	1.4121
10	164.28	160.99	6.6998	0.0922
11	169.24	165.86	0.9034	2.8477
12	190.93	187.11	0.2731	0.4900
13	201.94	197.90	4.6858	1.6905
14	206.56	202.43	0.1101	0.8722
15	216.56	212.23	0.0892	2.5991
16	220.56	216.15	0.1636	1.8062
17	230.06	225.46	0.2375	1.8595
18	257.10	251.96	0.0523	0.4710
19	276.26	270.74	0.3759	1.8587
20	281.31	275.68	2.0529	3.2108
21	284.69	278.99	16.1790	6.0462
22	319.68	313.28	4.8593	5.1099
23	325.98	319.46	5.0416	1.7955
24	328.64	322.07	4.9411	6.8883
25	357.24	350.10	5.5058	52.3733
26	386.83	379.09	15.6054	1.9460
27	408.36	400.19	1.6752	68.4657
28	422.32	413.88	0.5226	1.8152
29	441.77	432.93	11.9080	89.5658
30	443.93	435.05	12.4879	26.5435
31	473.70	464.23	6.9069	32.9302

32	476.61	467.08	0.4060	3.4513
33	479.38	469.80	0.3440	9.5713
34	504.36	494.28	82.4817	70.5084
35	531.25	520.62	2.4997	4.2420
36	547.67	536.72	1.2003	0.9816
37	562.73	551.48	3.6809	79.1102
38	578.37	566.80	47.2765	39.1375
39	587.26	575.51	0.9522	2.4521
40	602.62	590.57	7.9669	18.5754
41	618.56	606.19	11.8311	41.2871
42	644.10	631.22	0.3048	0.3575
43	662.57	649.32	0.0942	2.4123
44	700.84	686.82	39.6525	9.8549
45	706.48	692.35	14.9242	2.1388
46	719.07	704.69	10.0707	13.7942
47	742.38	727.53	2.0295	1.0745
48	764.04	748.76	0.6923	0.3676
49	775.24	759.74	53.3547	17.5906
50	799.82	783.82	2.1265	1.0374
51	823.46	806.99	26.3031	63.9010
52	831.65	815.02	9.4887	2.5558
53	842.39	825.54	101.3458	0.6760
54	854.44	837.35	33.0515	0.3756
55	875.96	858.44	60.8936	1.3375
56	885.47	867.76	22.7417	0.5487
57	896.91	878.97	75.7572	2.3213
58	911.62	893.39	1.0348	184.7337
59	950.90	931.88	7.7799	56.1927
60	978.61	959.04	40.6881	281.6260
61	1002.12	982.08	36.2909	20.1859
62	1020.68	1000.26	30.1540	91.3932
63	1037.50	1016.75	76.8046	33.9467
64	1060.87	1039.65	83.2720	48.8511
65	1062.01	1040.77	11.8680	4.7335
66	1080.33	1058.72	17.7436	29.8888
67	1150.78	1127.76	66.6321	385.9900
68	1162.26	1139.01	350.1704	66.1818
69	1170.74	1147.33	1.5996	1.8182
70	1170.78	1147.36	1.5558	2.1699
71	1180.87	1157.25	336.6937	219.3835
72	1207.36	1183.22	58.4117	130.3493
73	1222.78	1198.33	269.6139	70.8723
74	1227.05	1202.51	87.7433	77.5172
75	1237.69	1212.94	367.4016	22.8149
76	1260.53	1235.32	107.8725	806.9532
77	1277.32	1251.78	138.5619	1900.8554
78	1307.42	1281.27	201.8914	35.4254
79	1311.96	1285.72	771.6662	17.6965
80	1337.95	1311.19	1131.2534	96.0904
81	1366.24	1338.92	440.5375	14.9428
82	1381.50	1353.87	160.5041	138.8125
83	1390.54	1362.73	275.4767	1386.5043

84	1416.41	1388.08	2.5991	25.8460
85	1423.22	1394.75	314.6345	1548.8009
86	1444.36	1415.47	27.6117	207.0003
87	1453.18	1424.12	176.0962	78.1377
88	1466.62	1437.29	9.8492	19.4818
89	1469.29	1439.90	392.5704	529.2937
90	1472.65	1443.19	8.4808	21.0074
91	1485.30	1455.60	204.9386	17.4992
92	1490.12	1460.31	11.9988	23.3016
93	1491.22	1461.40	12.8706	28.0448
94	1494.28	1464.39	108.9480	1470.3811
95	1499.45	1469.46	29.8005	9.7744
96	1502.13	1472.08	10.5177	12.9055
97	1507.78	1477.63	16.4087	45.0881
98	1515.35	1485.04	295.9713	48.9679
99	1580.13	1548.53	374.5556	165.6257
100	1608.09	1575.92	22.1571	778.9838
101	1619.95	1587.55	88.1609	192.1476
102	1629.71	1597.12	196.2373	287.8391
103	1637.68	1604.93	111.0291	158.1742
104	1648.70	1615.73	848.3166	346.6863
105	1677.79	1644.24	72.4062	778.3749
106	1707.13	1672.99	586.5086	1079.3793
107	3023.40	2962.93	55.6923	224.6035
108	3025.90	2965.38	59.3136	342.5956
109	3045.19	2984.28	27.2091	337.1508
110	3088.53	3026.76	32.0550	86.2737
111	3092.53	3030.68	27.9667	120.0546
112	3101.84	3039.81	279.5780	791.5713
113	3104.43	3042.35	9.7530	116.4867
114	3113.90	3051.62	29.9641	182.8536
115	3148.00	3085.04	22.3849	209.0184
116	3153.94	3090.86	18.3108	204.1115
117	3185.25	3121.55	9.2618	310.2951
118	3217.87	3153.52	3.7048	321.1129
119	3228.37	3163.81	0.9609	133.9730
120	3236.87	3172.13	155.3493	826.9853
